# Closed-form stochastic solutions for non-equilibrium dynamics and inheritance of cellular components over many cell divisions

**DOI:** 10.1098/rspa.2015.0050

**Published:** 2015-08-08

**Authors:** Iain G. Johnston, Nick S. Jones

**Affiliations:** Department of Mathematics, Imperial College London, South Kensington Campus, London SW7 2AZ, UK

**Keywords:** stochastic processes, stochastic biology, cellular populations

## Abstract

Stochastic dynamics govern many important processes in cellular biology, and an underlying theoretical approach describing these dynamics is desirable to address a wealth of questions in biology and medicine. Mathematical tools exist for treating several important examples of these stochastic processes, most notably gene expression and random partitioning at single-cell divisions or after a steady state has been reached. Comparatively little work exists exploring different and specific ways that repeated cell divisions can lead to stochastic inheritance of unequilibrated cellular populations. Here we introduce a mathematical formalism to describe cellular agents that are subject to random creation, replication and/or degradation, and are inherited according to a range of random dynamics at cell divisions. We obtain closed-form generating functions describing systems at any time after any number of cell divisions for binomial partitioning and divisions provoking a deterministic or random, subtractive or additive change in copy number, and show that these solutions agree exactly with stochastic simulation. We apply this general formalism to several example problems involving the dynamics of mitochondrial DNA during development and organismal lifetimes.

## 1. Introduction

Stochastic dynamics underlie a multitude of processes in cellular biology [1–6]. Understanding the sources of this randomness within and between cells is a central current challenge in quantitative biology [7]. Noise has been found to affect processes including stem-cell fate decisions [8,9], bet-hedging in bacterial phenotypes [10,11], cancer development [12] and responses to apoptosis-inducing factors [13,14], illustrating the fact that a theoretical understanding of stochastic cellular biology is of great importance in medical and biological problems.

Partitioning of cellular components at cell divisions provides a considerable source of stochasticity in cell biology [15]. Huh and Paulsson [16] have shown that uneven segregation of cellular constituents at mitosis can contribute significantly to cell-to-cell differences in levels of cellular components and proteins in a population, focusing on stochasticity in protein inheritance between sister cells. In addition to variability in protein levels, there is evidence that random partitioning of mitochondria at cell divisions can lead to substantial extrinsic variability in the physical and chemical attributes, and behavioural phenomena including differentiation propensity, in a population of cells [17].

Historically, mathematical modelling, including the use of birth-and-death processes, has provided a theoretical foundation with which to describe phenomena in stochastic biology [18]. Early work on the problem of the stochastic evolution of cellular constituents in a population of dividing cells was performed in the context of protein levels in bacterial cells by Berg [19] and Rigney [20], who made analytic progress with birth-and-death models coupled to cell division in the case of a steady-state population of cells with constant birth-and-death rates. A famous example of stochastic analysis of cellular systems is the Luria–Delbrück treatment of population statistics of bacterial mutations [21], which has been addressed by several analyses including generating function approaches [22]. Matrix equations, with operators corresponding to the processes of birth, death and partitioning, have also been used to obtain numerical results on stochastic effects in populations of dividing cells [23,24], and a framework of non-equilibrium statistical mechanics has been used to derive general properties of protein content of dividing cells [25]. Stochastic models examining the behaviour of a continuous variable (for example, protein concentrations) have also been widely used in cellular biology [26].

Several stochastic models have been formulated for systems involving compartmentalized elements which replicate and are partitioned as compartments divide. An early example of this approach is due to Dowman [27], who considers mean copy number and extinction probability of cellular elements for specific birth, death and partitioning dynamics. Other applications are in the study of growing intestinal crypts, in which a collection of crypts, each containing a replicating quantity of stem cells, grow and divide according to a branching process [28], and in the stochastic corrector model, whereby a population of replicators with given concentrations of constituents divide and propagate [29]. More recently, Swain *et al.* [30] have derived analytic results describing stochastic gene expression in dividing cells after a steady limit cycle has been reached. Quantitative results, in terms of integrals over kinetic rate dynamics, have been obtained for stochastic gene expression where cellular components are binomially partitioned at cell divisions [31]. The variance resulting from more general partitioning dynamics of cellular components has also been addressed for single-cell divisions [15,16].

In this article, we focus on birth–immigration–death (BID) dynamics rather than the well-studied model dynamics of stochastic gene expression. With this framework, we attempt to provide a model for the stochastic dynamics of cellular components other than gene products; we particularly focus on mitochondrial DNA (mtDNA) in several examples. Populations of hundreds or thousands of mtDNA molecules are typically present in eukaryotic cells, replicating and degrading somewhat independently of the cell cycle. mtDNA encodes vital bioenergetic machinery, making it an important target for stochastic analysis. We will consider a variety of partitioning regimes and, where possible, an arbitrary number of cell divisions, and aim to derive closed-form generating functions describing the dynamics of our model cellular components. In so doing, we avoid assumptions about equilibrium behaviour and restrictions to lower-order moments of copy number distributions, aiming to produce a non-equilibrium theory to describe stochastic dynamics in full distributional detail, at arbitrary times during any cell cycle. Our specific consideration of mtDNA dynamics provides a theoretical framework with which a class of models, often analysed numerically through simulation, can be described analytically.

In the first section, we introduce our formalism and derive generating functions for BID dynamics with binomial partitioning and the inheritance of a deterministically or randomly reduced or increased complement of the parent cell's population. We next illustrate the exact agreement between our theory and stochastic simulation, and investigate several example biological questions, regarding the dynamics of mtDNA during a copy number bottleneck and over organismal lifetimes involving many cell divisions. We further harness the power afforded by a generating function approach to explore extinction probabilities in these systems, and also perform our analysis for a given number of cell divisions in which cellular components are deterministically partitioned, and randomly partitioned in clusters. Finally, we discuss the implications of our mathematical formalism for approaches to stochastic biology.

## 2. Model and analysis

In this section, we detail the approach we take to obtain generating functions describing the stochastic dynamics of cellular agents subject to BID dynamics and stochastic partitioning at cell divisions. We first illustrate how the generating function describing agent dynamics within a cell cycle is derived. We next consider how this solution may be extended over cell divisions, using an assumption (later shown to be true) about the functional form of the expression describing the inheritance of agents at cell divisions. We then show that this extended solution gives rise to an overall generating function containing factors that are the solutions to recursion relations, where each recursive step corresponds to a cell division. We obtain solutions for these recursion relations, thus yielding general generating functions for dynamics over arbitrarily many cell divisions. Finally, we validate our early assumption for several important specific cases of inheritance dynamics and derive the specific solutions in these cases.

### (a) Agents within a cell cycle

**Figure 1.**
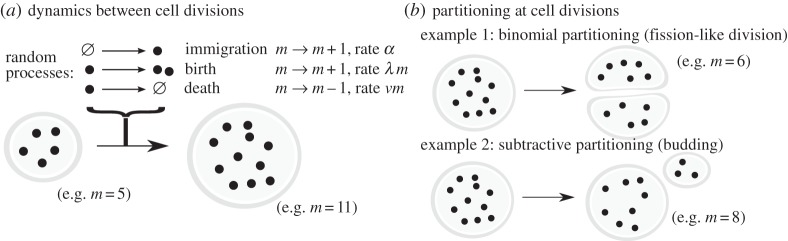
Illustration of birth–immigration–death (BID) dynamics with random partitioning. We will derive expressions for the statistics of copy number *m* of cellular agents over successive cell generations, separated by divisions. (*a*) Between cell divisions, agents may be produced, replicated or degraded; each is a Poissonian event. The copy number *m* of agents in a cell is a random variable that changes with these dynamics. (*b*) At cell divisions, the copy number of agents changes according to a different type of random event. Two possibilities are illustrated here: the binomial partitioning of agents into two daughter cells (one of which will be tracked in the next generation), and the loss of a random number of agents to a smaller bud (the larger remaining cell will be tracked in the next generation).

We consider BID dynamics, where agents are created (∅→•) with rate *α*, replicate (•→••) with rate λ and are removed (•→∅) with rate *ν* (figure 1*a*); these processes are referred to as immigration, birth and death, respectively, and are assumed to be Poissonian, with non-time-varying rates (see Results). We will later consider setting some of these parameters to zero, as special cases of the overall BID dynamics. We note that some literature refers to our ‘immigration’ term (producing agents from nothing) as ‘birth’, using the term ‘replication’ to describe the production of agents from existing agents; the specific symbols used to denote these rates also vary. For compatibility with a wide body of literature we adopt the nomenclature above.

The dynamics of populations under BID dynamics are given by the corresponding master equation, describing the time evolution of the probability *P*(*m*,*t*) that the system contains *m* agents at time *t*:

2.1 $$\frac{dP(m,t)}{dt}=\alpha P(m-1,t)+\nu (m+1)P(m+1,t)+\lambda (m-1)P(m-1,t)-(\alpha +\nu \;m+\lambda m)P(m,t),$$

with initial condition

2.2 $$P(m,0)=\delta_{mm_{0}}.$$

We are concerned with the generating function $G(z,t)=\sum_{m=0}^{\infty }z^{m}P(m,t)$ for the distribution of cellular components in a general set of conditions. Once this generating function has been calculated, then, by its definition, all information about the distribution *P*(*m*,*t*) can be obtained through taking its derivatives; for example, $E(m)=\partial G/\partial z{|}_{z=1}$, $V(m)=(\partial^{2}G/\partial z^{2}+\partial G/\partial z-{\left(\partial G/\partial z\right)}^{2}){|}_{z=1}$ and *P*(*m*)=(1/*m*!)(∂^*m*^*G*/∂*z*^*m*^)|_*z*=0_. The generating function corresponding to equations (2.1) and (2.2) obeys

2.3 $$\frac{\partial G(z,t)}{\partial t}=\alpha (z-1)G(z,t)+(\nu (1-z)+\lambda (z^{2}-z))\frac{\partial G(z,t)}{\partial z}$$

and

2.4 $$G(z,0)=z^{m_{0}},$$

the solution to which is well known (see electronic supplementary material, appendix S1):

2.5 $$\begin{array}{rl} G(z,t) & =\underset{\xi (z,t)}{\underbrace{{\left(\frac{\nu -\lambda }{\lambda \;e^{(\lambda -\nu )t}(z-1)-\lambda z+\nu }\right)}^{\alpha /\lambda }}}\;{\underset{g(z,t)}{\underbrace{\left(\frac{\nu \;e^{(\lambda -\nu )t}(z-1)-\lambda z+\nu }{\lambda \;e^{(\lambda -\nu )t}(z-1)-\lambda z+\nu }\right)}}}^{m_{0}} \end{array}$$

2.6 $$\begin{array}{r} \equiv \xi (z,t){\left(g(z,t)\right)}^{m_{0}}, \end{array}$$

where the final line, using the underbraced substitutions in equation (2.5), casts the solution in a form that will be useful later.

### (b) Partitioning of agents at cell divisions

We now consider a series of cell divisions, linked by quiescent periods governed by the within-cell-cycle dynamics above. Time is accounted for throughout this model in the following manner. *A full cell cycle is assumed to take time τ, after which a division occurs. A variable t Measures the elapsed time since the most recent cell division.* As divisions occur every interval *τ*, *t* is always less than or equal to *τ*. If *n* divisions have occurred, the total elapsed time since the initial state is *nτ*+*t*. We will here assume that *τ* is constant, showing later that this assumption can be relaxed when our approach is extended to deal with many different dynamic phases.

We will generally write *P*_*i*_(*m*,*t*|*m*_0_) for the probability of observing *m* agents at a time *t* after the *i*th cell division, given initial condition *m*_0_ (at the start of the cell cycle before the first cell division). Hence, *P*_0_(*m*,*t*|*m*_0_) is the probability of observing copy number *m* at time *t*, given that zero cell divisions have occurred; and *P*_*i*−1_(*m*,*τ*|*m*_0_) is the probability of observing *m* agents at a time *τ* after the (*i*−1)th cell division (i.e. immediately before the *i*th cell division).

Consider the *i*th cell division in a series of divisions. *Throughout this article, we will use subscript* *a* *to denote ‘after’ and subscript* *b* *to denote ‘before’ a cell division:* thus, we write *m*_*i*,*b*_ for copy number before the division and *m*_*i*,*a*_ for copy number afterwards. We assume that *m*_*i*,*b*_≥*m*_*i*,*a*_ always. We write *P*_*δ*_(*a*|*b*) for the probability of observing *a* agents after a cell division, given *b* agents before that division. The generating function at time *t* after the cell division is then given by

2.7 $$\begin{array}{rl} G_{i}(z,t) & =\sum_{m}\sum_{m_{i,b}=0}^{\infty }\sum_{m_{i,a}=0}^{m_{i,b}}z^{m}P_{0}(m,t|m_{i,a})P_{\delta }(m_{i,a}|m_{i,b})P_{i-1}(m_{i,b},\tau |m_{0}) \end{array}$$

2.8 $$\begin{array}{r} =\sum_{m_{i,b}=0}^{\infty }\sum_{m_{i,a}=0}^{m_{i,b}}G(z,t|m_{i,a})P_{\delta }(m_{i,a}|m_{i,b})P_{i-1}(m_{i,b},\tau |m_{0}) \end{array}$$

2.9 $$\begin{array}{r} =\sum_{m_{i,b}=0}^{\infty }\sum_{m_{i,a}=0}^{m_{i,b}}\xi (z,t)g{\left(z,t\right)}^{m_{i,a}}P_{\delta }(m_{i,a}|m_{i,b})P_{i-1}(m_{i,b},\tau |m_{0}). \end{array}$$

We will make the assumption that the expression in equation (2.9) may be reduced to the form

2.10 $$G_{i}(z,t)\equiv \xi (z,t)\phi (z,t)\sum_{m_{i,b}=0}^{\infty }\theta {\left(g(z,t)\right)}^{m_{i,b}}P_{i-1}(m_{i,b},\tau |m_{0}),$$

where *θ* and *ϕ* are functions to be determined, given knowledge of a particular partitioning rule. The partitioning mechanisms that we will subsequently consider can all be cast in this form, as we will demonstrate.

We now consider the overall generating function describing a set of cell divisions. To represent the sum over all possible copy numbers before and after all cell divisions between divisions *j* and *i*, we introduce the notation

2.11 $$\sum_{i,j}^{\prime }\equiv \sum_{m_{i,b}=0}^{\infty }\sum_{m_{i,a}=0}^{m_{i,b}}\sum_{m_{i-1,b}=0}^{\infty }\sum_{m_{i-1,a}=0}^{m_{i-1,b}}\cdots \sum_{m_{j,b}=0}^{\infty }\sum_{m_{j,a}=0}^{m_{j,b}}.$$

This combination of sums takes into account all possible states before and after each cell division *i*,*i*−1,…,*j*, for *i*≥*j*. We note that the ordering of sums here progresses backwards in time from left to right: the leftmost two sums sum over all configurations related to the most recent cell division *i*, the next two sum over all configurations related to the preceding cell division *i*−1, and so on. The final probability distribution is then

2.12 $$P_{n}(m,t|m_{0})=\sum_{n,1}^{\prime }P_{0}(m,t|m_{n,a})\prod_{i=1}^{n-1}\Phi_{i},$$

where *Φ*_*i*_ is a ‘probability propagator’ of the form

2.13 $$\Phi_{i}=P_{\delta }(m_{i,a}|m_{i,b})P_{0}(m_{i,b},\tau |m_{i-1,a}),$$

representing the probability that a cell, which started with *m*_*i*−1,*a*_ units after division *i*−1, grew to have *m*_*i*,*b*_ units, of which *m*_*i*,*a*_ units were inherited by the next daughter cell after division *i*. The chain of divisions can be terminated at *n* divisions in the past by setting *m*_0,*a*_≡*m*_0_ as the initial condition of the ancestor cell. Thus, a subscript 1 labels the first cell division, and a subscript *n* labels the most recent of *n* cell divisions.

The overall generating function after *n* divisions is

2.14 $$\begin{array}{rl} G_{n}(z,t|m_{0}) & =\sum_{m}\sum_{n,1}^{\prime }z^{m}P_{0}(m,t|m_{n,a})\prod_{i=1}^{n-1}\Phi_{i} \end{array}$$

2.15 $$\begin{array}{r} =\sum_{n,1}^{\prime }G(z,t|m_{n,a})\prod_{i=1}^{n-1}\Phi_{i} \end{array}$$

2.16 $$\begin{array}{r} =\sum_{n-1,1}^{\prime }\sum_{m_{n,b}=0}^{\infty }\sum_{m_{n,a}=0}^{m_{n,b}}\xi (z,t)g{\left(z,t\right)}^{m_{n,a}}\;P_{\delta }(m_{n,a}|m_{n,b})P_{0}(m_{n,b},\tau |m_{n-1,a})\prod_{i=1}^{n-2}\Phi_{i}. \end{array}$$

Generalizing the approach of Rausenberger & Kollmann [31], we now employ the assumption in equation (2.10) to write

2.17 $$\begin{array}{rl} & \sum_{n-1,1}^{\prime }\sum_{m_{n,b}=0}^{\infty }\sum_{m_{n,a}=0}^{m_{n,b}}\xi (z,t)g{\left(z,t\right)}^{m_{n,a}}P_{\delta }(m_{n,a}|m_{n,b})P_{0}(m_{n,b},\tau |m_{n-1,a})\prod_{i=1}^{n-2}\Phi_{i} \end{array}$$

2.18 $$\begin{array}{r} \;=\xi (z,t)\phi (z,t)\sum_{n-2,1}^{\prime }\sum_{m_{n,b}=0}^{\infty }\theta {\left(z,t\right)}^{m_{n,b}}P_{0}(m_{n,b},\tau |m_{n-1,a})\prod_{i=1}^{n-2}\Phi_{i} \end{array}$$

2.19 $$\begin{array}{rl} & \;=\xi (z,t)\phi (z,t)\sum_{n-2,1}^{\prime }G(\theta (g(z,t)),\tau |m_{n-1,a})\prod_{i=1}^{n-2}\Phi_{i} \end{array}$$

2.20 $$\begin{array}{r} \;\equiv \xi (z,t)\phi (z,t)\sum_{n-2,1}^{\prime }G(z_{1},\tau |m_{n-1,a})\prod_{i=1}^{n-2}\Phi_{i}, \end{array}$$

where in equation (2.20) we have changed variables *z*_1_≡*θ*(*g*(*z*,*t*)). Comparing equation (2.15) and (2.20), we can see that this process can be followed inductively. Each further step through the induction corresponds to another cell division, extracts a prefactor of *ϕ*(*z*_*i*_,*τ*), and causes another change of variables *z*_*i*+1_=*θ*(*g*(*z*_*i*_,*τ*)). Extending this induction to *n* cell divisions, the overall generating function after *n* divisions is then

2.21 $$\begin{array}{c} G_{n}(z,t)=\left(\prod_{i=1}^{n}\xi (z_{i},\tau )\right)\left(\prod_{i=1}^{n}\phi (z_{i},\tau )\right)\xi (z,t)h_{0}{\left(z,t\right)}^{m_{0}}, \end{array}$$

where *h*_*i*_ and *z*_*i*_ obey recurrence relations

2.22 $$h_{i}(z,t)=g(\theta (h_{i+1}),\tau );\;h_{n}(z,t)=g(z,t)$$

and

2.23 $$z_{i+1}=\theta (g(z_{i},\tau ));\;z_{1}=\theta (g(z,t)).$$

The recurrence relations equations (2.22) and (2.23) are rather similar, but we retain their distinction for mathematical convenience, also noting that their indexing runs in opposite directions through time. Hence, the boundary condition for *h*_*i*_ arises from the most recent cell division, corresponding to *i*=*n*; the boundary condition for *z*_*i*_ also arises from the most recent division, but in this indexing this corresponds to *i*=1. Of equation (2.22), only *h*_0_ plays a role in equation (2.21); from equation (2.23), all *z*_*i*_ for *i*=1 to *n* feature.

It can be noted that, for *n*=0, the products in equation (2.21) vanish and the boundary condition *h*_*n*_≡*h*_0_=*g*(*z*,*t*) leads to *G*_0_(*z*,*t*)=*ξ*(*z*,*t*)*g*(*z*,*t*)^*m*_0_^ as required.

For BID dynamics, the products and final terms in equation (2.21) are analytically tractable for several important inheritance regimes, allowing us to write the generating function in an exact form. We will first analyse two partitioning regimes of importance for biological modelling, and illustrate how this approach produces statistics of interest for mtDNA populations under these regimes. We will later explore other partitioning regimes.

### (c) Binomial inheritance

We first consider the case where agents are partitioned binomially at cell divisions. In this case, the following identities hold:

2.24 $$\begin{array}{rl} P_{\delta }(m_{i,a}|m_{i,b}) & =\left(\begin{array}{c} m_{i,b}\\ m_{i,a} \end{array}\right)2^{-m_{i,b}}; \end{array}$$

2.25 $$\begin{array}{rl} \sum_{m_{i,a}=0}^{m_{i,b}}\xi (z,t)g{\left(z,t\right)}^{m_{i,a}}P_{\delta }(m_{i,a}|m_{i,b}) & =\xi (z,t){\left(\frac{1}{2}+\frac{g(z,t)}{2}\right)}^{m_{i,b}}; \end{array}$$

and so

2.26 $$\begin{array}{rl} \phi (z,t) & =1; \end{array}$$

2.27 $$\begin{array}{rl} \theta (g(z,t)) & =\left(\frac{1}{2}+\frac{g(z,t)}{2}\right), \end{array}$$

where equations (2.26) and (2.27) follow by comparing equation (2.25) with equation (2.10). We are thus concerned with the solutions to the recurrence relations

2.28 $$z_{i}=\frac{1}{2}+\frac{g(z_{i-1},\tau )}{2};\;z_{1}=\frac{1}{2}+\frac{g(z,t)}{2}$$

and

2.29 $$h_{i}=g\left(\frac{1}{2}+\frac{h_{i+1}}{2},\tau \right);\;h_{n}=g(z,t).$$

We will introduce the symbols *l*≡e^(λ−*ν*)*τ*^ and *l*′≡e^(λ−*ν*)*t*^ for convenience here and throughout. In the electronic supplementary material, appendix S1, we solve these related systems of equations, showing that the solutions take the form

2.30 $$h_{i}=\frac{\kappa_{11}2^{i}+\kappa_{12}l^{i}}{\kappa_{13}2^{i}+\kappa_{14}l^{i}}$$

and

2.31 $$z_{i}=\frac{\kappa_{21}2^{i}+\kappa_{22}l^{i}}{\kappa_{23}2^{i}+\kappa_{24}l^{i}},$$

with *κ*_11_=*l*^*n*^*l*′(*z*−1)(λ+*ν*(*l*−2)), *κ*_12_=*κ*_14_=2^*n*^(λ(*l*′−*z*(*l*+*l*′−2))+*ν*(*l*−2)), *κ*_13_=*l*^*n*^*l*′(*z*−1)λ(*l*−1), *κ*_21_=*κ*_23_=−*l*(λ*l*′(*z*−1)+(*l*−2)(λ*z*−*ν*)), *κ*_22_=*l*′(*z*−1)(*l*(λ+*ν*)−2*ν*) and *κ*_24_=2λ*l*′(*z*−1)(*l*−1).

We also show in the electronic supplementary material, appendix S1, that the first product in equation (2.21) takes the form

2.32 $${\left(\frac{B_{1}^{n+1}B_{2}^{-n-1}(A_{2}+B_{2}){\left(-A_{1}/B_{1};\rho_{A}/\rho_{B}\right)}_{n+1}}{(A_{1}+B_{1}){\left(-A_{2}/B_{2};\rho_{A}/\rho_{B}\right)}_{n+1}}\right)}^{\gamma },$$

where *A*_1_=2λ*l*′(*l*−1)(*z*−1), *A*_2_=λ*ll*′(*z*−1)(*l*−1), *B*_1_=*B*_2_=*l*(λ*l*′(1−*z*)−(*l*−2)(λ*z*−*v*)), *ρ*_*A*_=*l*, *ρ*_*B*_=2 and *γ*=*α*/λ. (*a*;*q*)_*n*_ is the *q*-Pochhammer symbol defined by ${\left(a;q\right)}_{n}\equiv \prod_{k=0}^{n-1}(1-aq^{k})$. While this symbol is hard to interpret intuitively, we will see that expressions for important moments of distributions often only involve particular derivatives of the symbol that reduce to simple algebraic expressions (see Results). In addition, this term vanishes in the *α*=0 case where immigration dynamics can be ignored.

**Figure 2.**
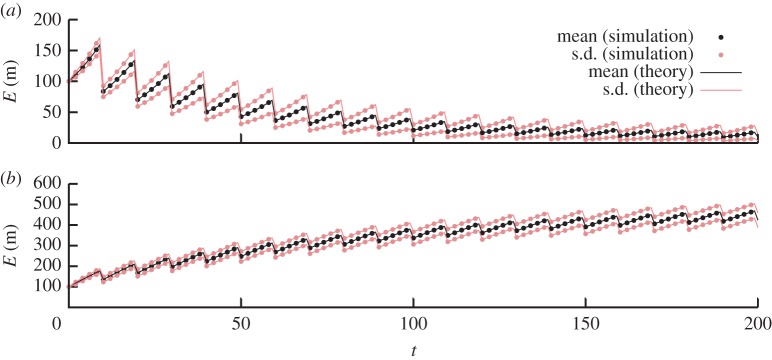
Comparison of analytic results for the first two moments with stochastic simulation. Trajectories of copy number mean and standard deviation resulting from our analytic results (lines) and stochastic simulation (points). (*a*) Birth–immigration–death (BID) dynamics within cell cycles; binomial partitioning at cell divisions. (*b*) BID dynamics within cell cycles; loss of a binomially distributed number of agents ($p=\frac{1}{2},\;N=100$) at each cell division. Other parameters for these systems are given in the text. There is an excellent match between theory and simulation in all cases.

Combining *h*_0_ from equation (2.30) and equation (2.32), we can then use equation (2.21) to yield a closed-form generating function for BID dynamics with binomial partitioning at cell divisions (the full form is explicitly presented in the electronic supplementary material, appendix S1). In the Results section, we will demonstrate the efficacy of this generating function solution (and subsequent solutions) by showing that moments derived from the generating function exactly match stochastic simulation (figure 2). This algebraic generating function for an arbitrary number of cell divisions extends a previous solution presented in integral form from [31], with the advantage that the methodology allows straightforward analytic investigation of this and a wider class of systems; we next demonstrate this generalization with a new inheritance regime which we will demonstrate has biological applicability.

### (d) Random additive or subtractive inheritance

We now consider the case where a number of agents are lost or gained at each cell division, and this number is itself a random variable (we will consider the case where this number is a fixed constant later). For mathematical convenience, we shall first assume that a certain number of agents are lost at partitioning and that this number is taken from a binomial distribution with population size 2*η* and probability $\frac{1}{2}$, so that the average loss number is *η* (it is straightforward to see that a negative value for the *η* parameter corresponds to the gain of a number of elements identically distributed). In this case, by considering the possible values of *n*, the number of agents lost at a division, we obtain

2.33 $$\begin{array}{rl} P_{\delta }(m_{i,a}|m_{i,b}) & =\sum_{n=0}^{2\eta }\delta_{m_{i,a},m_{i,b}-n}\left(\begin{array}{c} 2\eta \\ n \end{array}\right)2^{-2\eta }; \end{array}$$

2.34 $$\begin{array}{rl} \sum_{m_{i,a}=0}^{m_{i,b}}\xi (z,t)g{\left(z,t\right)}^{m_{i,a}}P_{\delta }(m_{i,a}|m_{i,b}) & =\xi (z,t){\left(\frac{1}{2}+\frac{1}{2\;g(z,t)}\right)}^{2\eta }g{\left(z,t\right)}^{m_{i,b}}; \end{array}$$

and so

2.35 $$\begin{array}{rl} \phi (z,t) & ={\left(\frac{1}{2}+\frac{1}{2\;g(z,t)}\right)}^{2\eta }; \end{array}$$

2.36 $$\begin{array}{rl} \theta (g(z,t)) & =g(z,t). \end{array}$$

The solutions to the corresponding recurrence relations are derived in the electronic supplementary material, appendix S1, and are

2.37 $$h_{i}=\frac{l^{n}l^{\prime }\nu (z-1)+l^{i}(\nu -\lambda z)}{l^{n}l^{\prime }\lambda (z-1)+l^{i}(\nu -\lambda z)}$$

and

2.38 $$z_{i}=\frac{l^{i}l^{\prime }\nu (z-1)+l(\nu -\lambda z)}{l^{i}l^{\prime }\lambda (z-1)+l(\nu -\lambda z)}.$$

The first product in equation (2.21) is $\prod_{i=1}^{n}\xi (z_{i},\tau )$, which we show in the electronic supplementary material, appendix S1, takes the form of equation (2.32) with *A*_1_=λ*l*′(*z*−1), *A*_2_=λ*ll*′(*z*−1), *B*_1_=*B*_2_=*l*(*ν*−λ*z*), *ρ*_*A*_=*l*, *ρ*_*B*_=1 and *γ*=*α*/λ. The second product term is $\prod_{i=1}^{n}{\left(1/2+1/(2g(z_{i},\tau ))\right)}^{2\eta }$. We here introduce the symbols *x*_1_≡λ(*l*^−1^−1)/(λ−*ν*) and *x*_2_≡λ(*l*−1)/(λ−*ν*). In the electronic supplementary material, appendix S1, we show that this product takes the form of equation (2.32) with *A*_1_=*l*′(λ+*ν*)(−*x*_2_)^*n*^(*z*−1), $B_{1}=B_{2}=-2x_{1}^{n}(\lambda z-\nu )$, *A*_2_=2*l*′*ν*(−*x*_2_)^*n*^(*z*−1), *ρ*_*A*_=*x*_1_, *ρ*_*B*_=(−*x*_2_) and *γ*=2*η*.

We then have a closed-form generating function for random subtractive partitioning and BID dynamics (the full form is presented in the electronic supplementary material, appendix S1). We note here that applying this approach to the case of loss at cell divisions does not explicitly restrict copy number to be non-negative, and care must therefore be taken in its application. If the dynamics under investigation are such that the probability of copy number *m*<*η* is negligible, the absence of this restriction will have negligible influence on results extracted from the analysis. If low copy numbers are likely, this approach can still yield useful results if *α*=0, if expressions for *P*(*m*,*t*) are derived and *P*(*m*≤0,*t*) is treated as equivalent to *P*(*m*=0,*t*). This approximation is valid because the birth and death operations have *m*=0 as an absorbing state, so a copy number below zero can never subsequently exceed *m*=0. However, the simple expressions for E(m,t) and V(m,t) in terms of generating function derivatives will yield incorrect results in these cases. The approximation will fail in cases where a non-negligible probability of attaining zero copy number is coupled with dynamics involving immigration (as opposed to birth). In this case, a specific boundary rule must be written in equation (2.1); we have been unable to find closed-form solutions in this case.

## 3. Results and applications

### (a) Comparison with stochastic simulation

In figure 2, we compare the analytic results for copy number mean and variance, derived from our generating functions above, with the results obtained over 10^5^ simulations using Gillespie's stochastic simulation algorithm [32]. In order to compute moments arising from the generating functions for subtractive inheritance it is necessary to compute a small number of values corresponding to derivatives of the *q*-Pochhammer symbol (*a*;*q*)_*n*_ with respect to the first argument *a*. These values can be evaluated to arbitrary precision by symbolic software or, if such software is not available to the reader, through numerical perturbation, by evaluating ((*a*;*q*)_*n*_−(*a*+*ϵ*;*q*)_*n*_)/*ϵ* for suitably small *ϵ*. In this case, we take ‘suitably small’ to mean ‘yielding an estimate converged to the required degree of accuracy’. In addition, in several cases which arise (e.g. *a*=0, emerging from our analysis below), this perturbative approach yields an analytic solution. In the electronic supplementary material, we provide Mathematica notebooks illustrating these calculations (and other calculations in this article).

We choose arbitrary parametrizations for these confirmatory studies: we use *m*_0_=100,*τ*=10, for binomial partitioning we use *α*=0.2, λ=0.06, *ν*=0.01, and for subtractive inheritance we use *α*=10, λ=0.01, *ν*=0.02, with *η*=50 for deterministic and random subtraction. Analytic results provide an excellent match to simulation results throughout.

We proceed by considering two examples motivated by specific biological questions involving cellular populations of mtDNA.

### (b) mtDNA bottlenecking: birth dynamics, binomial partitioning, changing population size

mtDNA is observed to be present in fertilized oocytes at copy numbers around 10^5^ [33–35]. During subsequent development, a pronounced copy number decrease occurs, as cells divide rapidly with little replication of mtDNA. The copy number per cell falls to a low bottleneck then recovers during later development. This mechanism is believed to ameliorate the inheritance of mutated mtDNA by increasing the cell-to-cell variability of mutant load in a cellular population and hence allowing cell-level selection to discard those cells that drift towards high mutant content. Substantial debate surrounds this topic [36,37]: competing mechanisms have been proposed to increase mutant load variability [34,35], and the size of the copy number bottleneck, its power to generate variance, and thus its biological importance have been questioned [34].

Results from classical population genetics [38,39] have been applied to the statistics of mtDNA populations [40], but even with refinements modelling fluctuations in the size of, and substructure in, the mtDNA population [41,42], these results lack straightforward physical interpretability and the ability to address population statistics at arbitrary, non-steady-state points in developmental dynamics. The stochastic formalism we present has recently been used to address these issues, specifically in modelling the mtDNA bottleneck in mice [43]. Here we explore a more general question: what increase in copy number variance is possible through enforcing an mtDNA bottleneck of a specific size, and how does it relate to the size of that bottleneck? Results on the copy number statistics of mtDNA can then readily be extended to explore statistics of the mutant load of mtDNA, by considering two decoupled populations of mutant and wild-type mtDNA.

In the case of the birth–death (BD) model with binomial partitioning, we can explore the levels of cellular noise introduced by controlled variation of the population size of cellular components without making continuous or steady-state approximations. We will consider a number *r*_max_ of dynamic phases labelled by *r*, where the rates λ,*ν* are constant within a phase but may take different values in different phases. To extend the above reasoning to describe different phases of dynamics it is necessary to compute the function *g*_*r*_(*z*,*t*) for each regime *r*, where *g*_*r*_(*z*,*t*) is the generating function using the appropriate parameters λ_*r*_, *ν*_*r*_ for phase *r* and calculated at *n*=*n*_*r*_, the number of cell cycles of phase *r*. For consistency with the above approach, we label phases starting from a zero index, so the first phase corresponds to *r*=0, and we use $r_{\max}$ to denote the label of the final phase. Then we use

3.1 $$\begin{array}{rl} h_{r_{\max}} & =g_{r_{\max}}(z,t), \end{array}$$

3.2 $$\begin{array}{rl} h_{r} & =g_{r}(h_{r+1},0) \end{array}$$

3.3 $$\begin{array}{rl} \;\mathrm{and}\;G_{\mathrm{overall}} & =h_{0}^{m_{0}}, \end{array}$$

using induction over the different phases in the way we used induction over different cell cycles above. Here we consider the changeover between phases by using the generating function at the start of the incoming phase.

The system equations (3.1)–(3.3) can be solved for arbitrarily many phases with different kinetic parameters, producing closed-form results for a wide range of different dynamic trajectories, including arbitrarily varying population size and cell doubling times (see electronic supplementary material, appendix S1). We illustrate this approach with the following simple two-phase model system. Initially, *m*_0_ agents exist in a cell, with no associated cell-to-cell variability. These agents subsequently follow birth-only dynamics and binomial partitioning at cell divisions. The rate of birth is initially λ_1_ in the first dynamic phase, changing to λ_2_ after *n*_1_ cell divisions. We choose $\lambda_{2}=2\ln2/\tau -\lambda_{1}$, to ensure that mean copy number returns to *m*_0_ after a further *n*_1_ cell divisions. We will use $\lambda_{2}\geq \lambda_{1}\leq \ln2/\tau$, so the mean copy number either remains constant or initially drops to a minimum (the ‘bottleneck’) before recovering.

For simplicity, we have here assumed that *τ*, cell-cycle length, is the same constant in each dynamic phase. This assumption can readily be relaxed by using different *τ*_*i*_, so that cell-cycle length is labelled by the current dynamic phase. In this way, our formalism can be used to explore the dynamics of systems with arbitrarily varying (though deterministic) cell-cycle lengths.

Using equation (2.21) with equations (2.30) and (2.32), the solution to equations (3.1)–(3.3) for two dynamic phases is simply *G*=*g*_1_(*g*_2_(*z*,*t*),0)^*m*_0_^, with *α*=0 and *ν*=0. After some algebra, we obtain, after *n*_1_ cell divisions in the first phase and another *n*_1_ in the second,

3.4 $$V(m)=\frac{m_{0}\;e^{-\lambda_{1}n_{1}\tau }(e^{\lambda_{1}\tau }+2)(e^{\lambda_{1}n_{1}\tau }-2^{n_{1}})}{e^{\lambda_{1}\tau }-2}.$$

The minimum copy number attained immediately follows cell division *n*_1_ and is thus of size *b*=2^−*n*_1_^*m*_0_ e^*n*_1_λ_1_*τ*^. This allows us to write the parameter λ_1_ in terms of the bottleneck it produces, $\lambda_{1}=\ln(2^{n_{1}}b/m_{0})/(n_{1}\tau )$. Inserting this expression into equation (3.4) gives

3.5 $$V(m)=\frac{m_{0}(b-m_{0})}{b}\left(\frac{{\left(b/m_{0}\right)}^{1/n_{1}}+1}{{\left(b/m_{0}\right)}^{1/n_{1}}-1}\right).$$

**Figure 3.**
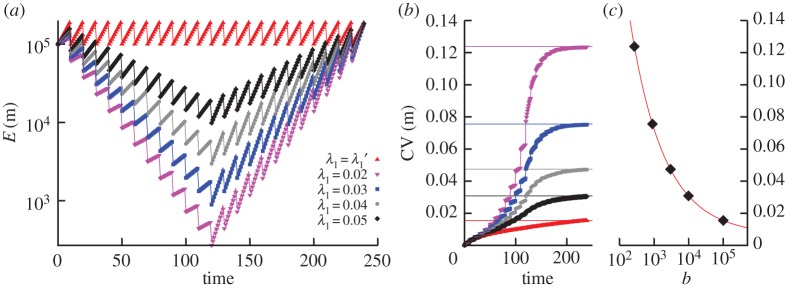
Modelling copy number variability due to mtDNA bottlenecking. (*a*) Trajectories of mean copy number of cellular agents born with rate λ_1_ (*t*<120) and $\lambda_{2}=2\ln2/\tau -\lambda_{1}$ (*t*≥120). Lower λ_1_ values enforce a smaller copy number bottleneck, with copy number recovering to its initial value after the bottleneck. $\lambda_{1}^{\prime }=\ln2\tau$ is the value required to maintain a constant-mean copy number between cell divisions. Lines are analytic results; points are stochastic simulations. (*b*) Trajectories of coefficient of variation (CV) as different bottleneck sizes are imposed on the system. Horizontal lines give the analytic predictions for final CV derived from equation (3.5). Other lines are analytic results; points are stochastic simulations. (*c*) CV as a function of bottleneck size from equation (3.5); points show specific instances of stochastic simulation.

In figure 3, we simulate the above system for *n*_1_=12, *m*_0_=10^5^, roughly matching the above mtDNA copy number magnitudes and number of divisions [44] observed in the mouse germ line. We use various λ_1_ values, reporting mean copy number and copy number coefficient of variation (CV). It can be observed that lower λ_1_ rates lead to more pronounced copy number bottlenecks, resulting in increased CVs that match the predictions derived from the above analysis.

Equation (3.5) analytically describes the increased variance due to a copy number bottleneck under specific circumstances, whereby a copy number *m*_0_ is decreased to a minimum *b* over *n*_1_ cell divisions then raised to its original value over a further *n*_1_ cell divisions. Lower bottleneck sizes *b* lead to exponential increases in the cell-to-cell variability associated with an mtDNA population. The assumptions within our illustrative model here can also straightforwardly be relaxed and closed-form solutions derived for more general dynamics, and a closed-form expression for the probability distribution function can also be derived using the above approach. These results can then be used to address any statistical questions about the system, including fixation probabilities of mutant mtDNA (readily extracted from the generating function) and the behaviour of heteroplasmy variance with time (from the variances of mutant and wild-type populations). For brevity we here just present these illustrative results for copy number variance; further development of a more specific model is a focus of [43]. We note that, in this example, the variance does not converge to a final fixed value: longer times will result in higher variances. This feature can be altered using a model involving more homeostatic dynamics (see next subsection) or, in biology, may conceivably be dealt with on a cellular level by retaining cells with certain copy number statistics.

### (c) Relaxed replication of mtDNA: immigration–death dynamics and various inheritance regimes

A quantitative model for mtDNA dynamics throughout organismal lifetimes has been proposed to account for the intuitive feature that mtDNA copy number should be subject to cellular control [45]. This ‘relaxed replication’ model has influenced a wide range of studies on mtDNA dynamics in many contexts from human disease [46] to forensics [47]; its quantitative behaviour has been explored (considering low-order moments without cell divisions) in the contexts of nuclear control on mtDNA [48] and through simulation studies in topics including ageing mtDNA [49], the effect of anti-retroviral drugs on mtDNA [50], and many others. As variability in mtDNA can have important physiological consequences [36], we aim here to analytically extend this model beyond a mean-only treatment to a more general (and realistic) situation both explicitly modelling the stochastic dynamics of individual mtDNA production and degradation and including different forms of cell division dynamics.

The governing dynamics of copy number *m* in the model are

3.6 $$\frac{dm}{dt}=\left\{ \begin{array}{ll} \frac{\tilde{\alpha }m_{\mathrm{opt}}}{\tilde{\tau }}-\frac{\tilde{\alpha }m}{\tilde{\tau }} & \text{if}\;m\leq \frac{\tilde{\alpha }m_{\mathrm{opt}}}{\tilde{\alpha }-1};\\ -\frac{m}{\tilde{\tau }} & \text{otherwise,} \end{array}\right.$$

where *m*_opt_ is a ‘target’ copy number, $\tilde{\tau }$ is the time scale of mtDNA degradation, and $\tilde{\alpha }>1$ is a parameter of the model describing nuclear feedback (in the original papers, $\tilde{\alpha }$ and $\tilde{\tau }$ are, respectively, assigned the symbols *α* and *τ*: we use tildes to avoid ambiguity with the symbols in our preceding analysis). For $\tilde{\alpha }$ not much greater than 1 and an initial condition *m*_0_<*m*_opt_, the probability of $m>\tilde{\alpha }m_{\mathrm{opt}}/(\tilde{\alpha }-1)$ is very low; we will thus assume that the contribution of the term in equation (3.6) corresponding to $m>\tilde{\alpha }m_{\mathrm{opt}}/(\tilde{\alpha }-1)$ is negligible.

When considering cell divisions in the above model, the meaning of *m*_opt_ needs to be made explicit. We will consider *m*_opt_ to be the target copy number for the end of a cell cycle, immediately before division. We then note that the first term in equation (3.6) contains a combination of an immigration term (independent of copy number) and a death term (dependent on copy number); we can therefore write the model as equation (2.1) with *α*≡*βm*_opt_, λ=0 and *ν*≡*β*, defining the new parameter $\beta \equiv \tilde{\alpha }/\tilde{\tau }$. We can then use the above treatment to obtain the generating function of the relaxed replication model under different partitioning regimes (full forms are given in the electronic supplementary material, appendix S1) and moments of interest of the copy number distribution in each case.

**Figure 4.**
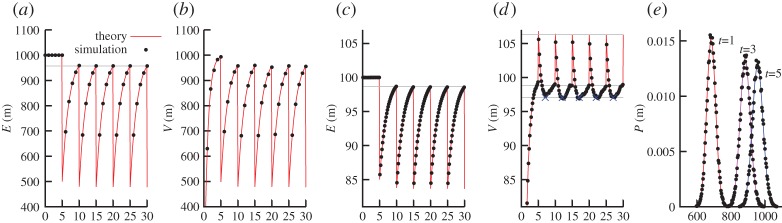
Copy number statistics for the relaxed replication model of mtDNA. Lines are analytic results; points are from stochastic simulation. (*a*) E(m) and (*b*) V(m) for binomial partitioning, modelling dividing cells, with *m*_0_=*m*_opt_=1000, $\beta =\frac{1}{2}$. V(m) and E(m) converge to the same trajectory; the grey line gives the analytic result for E(m) at the end of a cell cycle. (*c*) E(m) and (*d*) V(m) for random subtractive partitioning, modelling budding cells, with *m*_0_=100=*m*_opt_=100, $\beta =\frac{1}{2}$, *η*=15. Grey lines give analytic results for E(m) at the end of a cell cycle, and V(m) at the start, end and minimum point (blue crosses) in the cell cycle. (*e*) Comparison of theory and stochastic simulation in illustrative snapshots of probability distribution functions at given times in the binomial case using equation (3.8).

*Binomial partitioning*. First we consider the case where mtDNA molecules are binomially partitioned at cell divisions. The dynamics in this case are illustrated in figure 4 for *m*_opt_=*m*_0_=1000, $\tilde{\alpha }=5$, $\tilde{\tau }=10$ and cell-cycle length *τ*=5. This parameter set was chosen for compatibility with original work on the relaxed replication model: mtDNA copy numbers around 1000 are biologically reasonable, $\tilde{\alpha }=5$ is an intermediate value of the nuclear feedback parameter explored in [48], and $\tilde{\tau }=10$ corresponds to an mtDNA half-life of 10ln⁡2≃7, compatible with the range (in days) of half-lives assumed in [48]. The cell-cycle length *τ*=5 was chosen both for rough biological applicability (corresponding to cells dividing every 5 days) and to illustrate transient behaviour of the model.

It can be observed that the variance and the mean converge on the same behaviour; the difference between the two is straightforwardly found to be $E(m)-V(m)=4^{-n}m_{0}\;e^{-2\beta (t+n\tau )}$, clearly decreasing to zero with cell divisions *n*. The mean copy number immediately before a division E(m,τ) takes a value that approaches, but does not reach, *m*_opt_. Some algebra (see electronic supplementary material, appendix S1) shows that this value, in the long time limit, is

3.7 $$E(m,\tau )=m_{\mathrm{opt}}-\frac{1}{2\;e^{\beta \tau }-1}m_{\mathrm{opt}}.$$

The generating function analysis also allows an expression to be derived for the probability distribution function for mtDNA copy number (see electronic supplementary material, appendix S1),

3.8 $$P(m,t)=\left(\frac{1}{m!}\right){\left(-b\right)}^{m}{\left(1-b\right)}^{m_{0}-m}\;e^{-a}U\left(-m,1-m+m_{0},\frac{a(b-1)}{b}\right),$$

where *U*(.,.,.) is the confluent hypergeometric function of the second kind (Tricomi's function [51]), $a=m_{\mathrm{opt}}(1-e^{-\beta t}+(2^{-n}\;e^{-\beta t}(e^{\beta \tau }-1)(2^{n}-e^{-\beta n\tau }))/(2\;e^{\beta \tau }-1))$ and *b*=2^−*n*^ e^−*β*(*t*+*nτ*)^. As n→∞, this converges to a simpler expression, neatly corresponding to a Poisson distribution:

3.9 $$P(m,t)=\frac{1}{m!}{\left(\frac{m_{\mathrm{opt}}(1-2\;e^{\beta \tau }+e^{-\beta (t-\tau )})}{1-2\;e^{\beta \tau }}\right)}^{m}\exp\left(\frac{-m_{\mathrm{opt}}(1-2\;e^{\beta \tau }+e^{-\beta (t-\tau )})}{1-2\;e^{\beta \tau }}\right).$$

Equation (3.8) thus provides a complete solution for the relaxed replication model with binomial cell divisions (see figure 4 for a comparison with stochastic simulation for E(m), V(m) and *P*(*m*); other statistics and quantities of interest are readily extracted from the generating function).

*Subtractive partitioning*. Next we consider the case of dynamics under which a random amount of mtDNA is lost at each division. This picture could model, for example, mtDNA dynamics in budding yeast, where cells with 50–200 mtDNA molecules [52] undergo asymmetric partitioning, with 10–20% of their mitochondrial content being lost at budding events, and homeostasis acting to maintain copy number [53]. Figure 4 illustrates the behaviour of this system with *m*_opt_=100, *η*=15. Interestingly in this case, the variance of copy number reaches a minimum at an intermediate point in each cell cycle, after the partitioning event (which increases the variance) and before an extended period of dynamics under homeostasis has led to an increase in copy number and variance. As above, the difference between *m*_opt_ and E(m,τ) can easily be computed in the long time limit (see electronic supplementary material, appendix S1):

3.10 $$E(m,\tau )=m_{\mathrm{opt}}-\frac{\eta }{e^{\beta \tau }-1}.$$

The expression for the variance in this case involves derivatives of the *q*-Pochhammer symbol, which at first would seem to prevent extracting simple and intuitive expressions for the variance. However, as with several applications of this approach, the derivatives involved all reduce to simple algebraic expressions (see electronic supplementary material, appendix S1). Taking the limit of many cell divisions n→∞, we obtain

3.11 $$V(m,t)=m_{\mathrm{opt}}+\frac{\eta }{2(e^{2\beta \tau }-1)}(3\;e^{2\beta (\tau -t)}-2\;e^{\beta (\tau -t)}-2\;e^{\beta (2\tau -t)}).$$

This expression allows us to characterize the form of the variance curve. Writing *η*′≡*η*/(2(e^2*βτ*^−1)), we find that $V(m,0)=m_{\mathrm{opt}}+\eta^{\prime }(e^{2\beta \tau }-2\;e^{\beta \tau })$ and $V(m,\tau )=m_{\mathrm{opt}}+\eta^{\prime }(1-2\;e^{\beta \tau })$, and that the minimum variance occurs at $t^{\prime }=\tau -(1/\beta )\ln(\frac{1}{3}(1+e^{\beta \tau }))$ and takes value $V(m,t^{\prime })=m_{\mathrm{opt}}-(\eta /6)\coth(\beta \tau /2)$. All these results are in agreement with stochastic simulation, as illustrated in figure 4, and other statistics and quantities of interest can readily be extracted from the generating function. We can thus characterize the expected biological behaviour of the relaxed replication model under different partitioning regimes, including quantities such as the probability of pronounced depletion of mtDNA (vanishingly small under these example parametrizations), and the cell-to-cell variability expected in a biological population.

We note that, as this example falls in the regime where *η*≪*m*_opt_, the probability of low copy number is negligible, so the statistics derived using our generating function approach are reliable (and the approach can also be used to compute the probability distribution function and other moments). In cases where low copy numbers are likely, caution must be used in employing this approach, as described above.

### (d) Extinction probabilities under balanced copy number dynamics

A strength of the use of generating functions to analyse stochastic dynamics and partitioning of cellular species is that statistics other than low-order moments can be straightforwardly computed. As demonstrated in the previous subsection, full probability distribution functions can be extracted for cellular populations of agents from generating functions, although these functions can be rather complicated. As simple examples of biological interest, we here consider the extinction probability *P*(0,*t*) of cellular species under two dynamic different regimes. The first is birth–death (BD) dynamics with binomial partitioning, with a ‘balancing’ choice of λ=ln⁡2/τ+ν, so that mean copy number stays constant through cell divisions. The second is the relaxed replication model above; that is, immigration–death (ID) dynamics with binomial partitioning, and *α*≡*βm*_opt_ and *ν*≡*β*. Both of these examples exhibit a balanced mean copy number, with the expected production of agents over a cell cycle balancing the expected loss through cell divisions. As previously described, the variance of the BD case increases with time, whereas the variance of the ID case converges.

Extinction probabilities can be straightforwardly extracted from our generating functions, using *P*(*m*=0)=*G*(0,*t*), allowing us to explore the probability of extinction under these dynamics. The resulting expressions under BD and ID models are

3.12 $$P_{\mathrm{BD}}(0,t)={\left(\frac{2^{t/\tau }(\nu \tau (n+2)+n\ln2)-2\nu \tau }{2^{t/\tau }(\nu \tau (n+2)+(n+2)\ln2)-2\nu \tau }\right)}^{m_{0}}$$

and

3.13 $$P_{\mathrm{ID}}(0,t)={\left(1-2^{-n}u^{n}u^{\prime }\right)}^{m_{0}}\exp\left(\frac{m_{\mathrm{opt}}(2^{-n}(u-1)u^{n}-(u-2)u^{\prime }-1)}{u^{\prime }(u-2)}\right),$$

where *u*≡e^−*βτ*^ and *u*′≡e^−*βt*^. Consideration of the n→∞ limit shows that, in the long time limit, extinction probability under the BD model converges to unity, whereas in the ID model a limiting probability is reached. Setting *t*=0 for simplicity (thus considering the population at the start of a cell cycle), this limiting probability is $\exp(-m_{\mathrm{opt}}(1+{\left(u-2\right)}^{-1}))$. The difference between the BD and ID cases is because of the irreversibility of extinction under the BD model (and the non-zero probability associated with extinction during every cell cycle); by contrast, extinction in the ID model can be escaped as immigration creates more agents in the cell without necessitating a non-zero source population. Hence, a non-zero probability flux away from the *m*=0 state exists, and eventually balances the flux into that state because of partitioning noise: the extinction probability may thus be thought of as representing the proportion of time during which the system occupies the *m*=0 state.

Further results can straightforwardly be extracted from our formalism for extinction probabilities in non-balanced cases. As a brief example, we consider the BD model with binomial partitioning, with a new parameter κ=λ−ν−ln⁡2/τ, so that *κ* measures the ‘excess’ birth rate beyond that required for copy number balance. For *κ*≤0, extinction is certain in the long time limit, but for *κ*>0 there is a finite probability that the copy number will never reach zero. To illustrate the qualitative behaviour of the system, we set *ν*=0, and we obtain

3.14 $$P_{\mathrm{BD}}(m=0)={\left(\frac{1-e^{\kappa n\tau }}{1+e^{\kappa n\tau }-2\;e^{\kappa (n+1)\tau }}\right)}^{m_{0}},$$

showing that extinction probability in the long time limit decays roughly exponentially with *κ*. The more general extinction probabilities for *ν*≠0 and in the absence of cell divisions are given in the electronic supplementary material, appendix S1.

### (e) Other inheritance dynamics

In addition to the binomial partitioning and random additive or subtractive changes of copy number, we have explored several other possible dynamic regimes of inheritance. The case where a fixed, deterministic number of agents is gained or lost at cell divisions is analytically tractable (see electronic supplementary material, appendix S1). We also consider deterministic halving of copy number, so that each daughter cell inherits half of the mother cell's content (rounded down). Additionally, we consider the inheritance of clusters of agents, such that agents are split into clusters of size *n*_c_ and these clusters are binomially partitioned. We have not found closed-form analytic solutions for the generating function for a general number of cell divisions for these latter two cases, but analytic statistics can nonetheless be obtained for a given number of cell divisions through the calculation of the appropriate recurrence relations (see electronic supplementary material, appendix S1).

**Figure 5.**
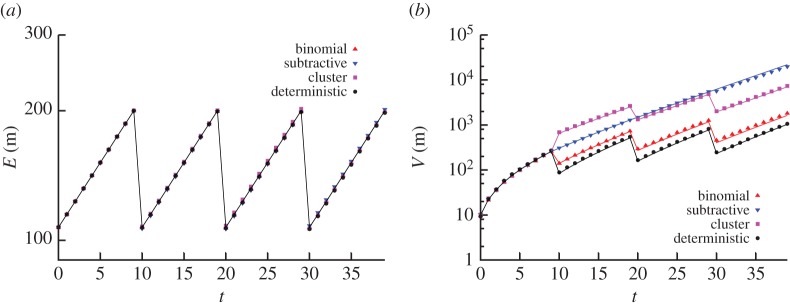
Solutions from recurrence relations for different partitioning regimes. Points are from stochastic simulation, lines are theoretical predictions. Deterministic, clustered, subtractive and binomial inheritance regimes are compared. Parameters used were *m*_0_=200, *ν*=0.01, *τ*=10, $n_{c}=10,\;\lambda =\log2/\tau +\nu$ and *η*=*m*_0_: the choice of the last two parameters was fixed to preserve a constant-mean copy number. Stochastic simulation results are from an ensemble of 10^5^ simulations of each situation.

Figure 5 illustrates the use of this approach to calculate the mean and variance of copy number for these systems, and for BD dynamics in the binomial and constant subtractive inheritance regimes described earlier. The agreement between stochastic simulation and analytic results is again excellent, showing, as expected, that deterministic inheritance leads to the lowest magnitude of stochasticity in copy number, followed by binomial partitioning, followed by clustered partitioning (illustrated for *n*_c_=10 in this case). We expect that other inheritance regimes of interest may be addressable through a similar approach.

## 4. Discussion

We have introduced a general mathematical formalism with which to address the stochastic dynamics of cellular agents that are inherited according to non-trivial and potentially stochastic dynamics at cell divisions. Our approach differs from, and extends, several previous tools designed to address stochastic partitioning in biology. First, our approach yields full, closed-form generating functions for several cases, allowing the extraction of all details of copy number distributions, rather than focusing on variance or other low-order moments alone. Second, we nowhere assume that a steady state or equilibrium has been reached, and are thus capable of extracting copy number statistics at any given time during the stochastic biological process of interest. Third, we focus on BID dynamics rather than the more common stochastic gene expression dynamics, with a view to modelling the behaviour of non-protein cellular components (including mtDNA, which we explore in particular). Fourth, we explore several specific partitioning regimes, obtaining closed-form results for arbitrary numbers of cell divisions under those we term binomial partitioning and random and deterministic subtractive or additive inheritance. We also obtain results for finite numbers of cell divisions under deterministic partitioning and binomial partitioning of clusters.

We have focused on agents undergoing BID dynamics between cell divisions; we expect that any random dynamics with a corresponding generating function of the form equation (2.6) will also admit treatment using this approach, paving the way for further generalization of this approach.

We note that the results arising from our analysis should be interpreted as ensemble statistics of single-cell measurements. If tracking a particular lineage of dividing cells, it should be remembered that the statistics of daughter cells will exhibit correlations because of their shared heritage. Our results represent expected statistics from a well-mixed bulk case.

In the absence of immigration dynamics, we have shown that a system governed by different rate parameters at different times can also be solved analytically. The rates associated with the production and destruction of cellular agents can vary arbitrarily as long as the rate of this change is lower than the cell division rate. This approach therefore provides a way to explore the statistics of stochastic systems with arbitrarily changing population size, in contrast with many results from classical statistical genetics which assume a constant or constant-mean population size (and additionally often only provide equilibrium results, preventing the quantitative exploration of systems before a steady state is reached) [38,39,41,42]. It is also straightforward to vary the cell-cycle length *τ* in these dynamic phases and so allow a treatment of cellular dynamics under varying division times. We have illustrated a general use of this approach in addressing the mtDNA bottleneck, and it has been used to explore the bottleneck specifically in mice in detail [43]. We believe that this formalism may prove useful in other contexts where organellar content is subject to dramatic and non-random population size changes; for example, in considering cellular populations during tumour development, where variability in cellular conditions causes time differences in physiological rate constants as tumour cells continually divide [54].

We have demonstrated the applicability of our stochastic formalism with some illustrative problems from cellular biology. We have found expressions for the cell-to-cell variability in mtDNA populations because of the imposition of a copy number bottleneck of given size, and extended the classic ‘relaxed replication’ model of mtDNA to include the stochastic dynamics of individual mtDNAs, and the effects of cell divisions. This model is widely influential in the study of mtDNA genetics and disease, but its quantitative analysis has typically been limited to descriptions of its mean behaviour or simulation studies with a fixed set of parameters. We have thus used our approach to further analytic understanding of this important model. Furthermore, we have explored in detail the statistics of populations of cellular agents under passive copy number balance over many cell divisions, a situation of importance for organelles and which may be of general applicability in cell biology.

Accurate models for the variability of cellular populations enable more powerful inference using experimental measurements of mean and variance across cells [55]. We hope that our results for stochastic inheritance dynamics will facilitate the strengthening of this link between theoretical and experimental biology and allow more information about underlying cellular dynamics to be obtained from the wealth of experimental measurements currently appearing.

## Supplementary Material

Appendix

## Supplementary Material

johnston-code.tar.gz

## Data accessibility

This article does not report any primary data. However, Mathematica notebooks and source code containing the analyses herein and illustrative calculations are available as the electronic supplementary material.

## References

[1] McAdams HH, Arkin A 1997 Stochastic mechanisms in gene expression. Proc. Natl Acad. Sci. USA 94, 814–819. (doi:10.1073/pnas.94.3.814) 9023339 10.1073/pnas.94.3.814 PMC19596

[2] Altschuler SJ, Wu LF 2010 Cellular heterogeneity: do differences make a difference? Cell 141, 559–563. (doi:10.1016/j.cell.2010.04.033) 20478246 10.1016/j.cell.2010.04.033 PMC2918286

[3] Elowitz MB, Levine AJ, Siggia ED, Swain PS 2002 Stochastic gene expression in a single cell. Science 297, 1183–1186. (doi:10.1126/science.1070919) 12183631 10.1126/science.1070919

[4] Kærn M, Elston TC, Blake WJ, Collins JJ 2005 Stochasticity in gene expression: from theories to phenotypes. Nat. Rev. Genet. 6, 451–464. (doi:10.1038/nrg1615) 15883588 10.1038/nrg1615

[5] Raj A, van Oudenaarden A 2008 Nature, nurture, or chance: stochastic gene expression and its consequences. Cell 135, 216–226. (doi:10.1016/j.cell.2008.09.050) 18957198 10.1016/j.cell.2008.09.050 PMC3118044

[6] Tsimring LS 2014 Noise in biology. Rep. Prog. Phys. 77, 026601 (doi:10.1088/0034-4885/77/2/026601) 24444693 10.1088/0034-4885/77/2/026601 PMC4033672

[7] Johnston IG 2012 The chaos within: exploring noise in cellular biology. Significance 9, 17–21. (doi:10.1111/j.1740-9713.2012.00586.x)

[8] Chang HH, Hemberg M, Barahona M, Ingber DE, Huang S 2008 Transcriptome-wide noise controls lineage choice in mammalian progenitor cells. Nature 453, 544–547. (doi:10.1038/nature06965) 18497826 10.1038/nature06965 PMC5546414

[9] Clayton E, Doupé DP, Klein AM, Winton DJ, Simons BD, Jones PH 2007 A single type of progenitor cell maintains normal epidermis. Nature 446, 185–189. (doi:10.1038/nature05574) 17330052 10.1038/nature05574

[10] Fraser D, Kærn M 2009 A chance at survival: gene expression noise and phenotypic diversification strategies. Mol. Microbiol. 71, 1333–1340. (doi:10.1111/j.1365-2958.2009.06605.x) 19220745 10.1111/j.1365-2958.2009.06605.x

[11] Kussell E, Kishony R, Balaban NQ, Leibler S 2005 Bacterial persistence: a model of survival in changing environments. Genetics 169, 1807–1814. (doi:10.1534/genetics.104.035352) 15687275 10.1534/genetics.104.035352 PMC1449587

[12] Brock A, Chang H, Huang S 2009 Non-genetic heterogeneity—a mutation-independent driving force for the somatic evolution of tumours. Nat. Rev. Genet. 10, 336–342. (doi:10.1038/nrg2556) 19337290 10.1038/nrg2556

[13] Bastiaens P 2009 Systems biology: when it is time to die. Nature 459, 334–335. (doi:10.1038/459334a) 19458703 10.1038/459334a

[14] Spencer SL, Gaudet S, Albeck JG, Burke JM, Sorger PK 2009 Non-genetic origins of cell-to-cell variability in TRAIL-induced apoptosis. Nature 459, 428–432. (doi:10.1038/nature08012) 19363473 10.1038/nature08012 PMC2858974

[15] Huh D, Paulsson J 2010 Non-genetic heterogeneity from stochastic partitioning at cell division. Nat. Genet. 43, 95–100. (doi:10.1038/ng.729) 21186354 10.1038/ng.729 PMC3208402

[16] Huh D, Paulsson J 2011 Random partitioning of molecules at cell division. Proc. Natl Acad. Sci. USA 108, 15 004–15 009. (doi:10.1073/pnas.1013171108) 10.1073/pnas.1013171108 PMC3169110 21873252

[17] Johnston IG, Gaal B, Neves RP, Enver T, Iborra FJ, Jones NS 2012 Mitochondrial variability as a source of extrinsic cellular noise. PLoS Comput. Biol. 8, e1002416 (doi:10.1371/journal.pcbi.1002416) 22412363 10.1371/journal.pcbi.1002416 PMC3297557

[18] Novozhilov AS, Karev GP, Koonin EV 2006 Biological applications of the theory of birth-and-death processes. Brief. Bioinform. 7, 70–85. (doi:10.1093/bib/bbk006) 16761366 10.1093/bib/bbk006

[19] Berg OG 1978 A model for the statistical fluctuations of protein numbers in a microbial population. J. Theor. Biol. 71, 587–603. (doi:10.1016/0022-5193(78)90326-0) 96307 10.1016/0022-5193(78)90326-0

[20] Rigney DR 1979 Stochastic model of constitutive protein levels in growing and dividing bacterial cells. J. Theor. Biol. 76, 453–480. (doi:10.1016/0022-5193(79)90013-4) 439915 10.1016/0022-5193(79)90013-4

[21] Luria SE, Delbrück M 1943 Mutations of bacteria from virus sensitivity to virus resistance. Genetics 28, 491–511. 17247100 10.1093/genetics/28.6.491 PMC1209226

[22] Zheng Q 1999 Progress of a half century in the study of the Luria–Delbrück distribution. Math. Biosci. 162, 1–32. (doi:10.1016/S0025-5564(99)00045-0) 10616278 10.1016/s0025-5564(99)00045-0

[23] Jones RB, Lumpkin CK Jr, Smith JR 1980 A stochastic model for cellular senescence. Part I. Theoretical considerations. J. Theor. Biol. 86, 581–592. (doi:10.1016/0022-5193(80)90354-9) 7218827 10.1016/0022-5193(80)90354-9

[24] Marshall WF 2007 Stability and robustness of an organelle number control system: modeling and measuring homeostatic regulation of centriole abundance. Biophys. J. 93, 1818–1833. (doi:10.1529/biophysj.107.107052) 17496020 10.1529/biophysj.107.107052 PMC1948063

[25] Brenner N, Shokef Y 2007 Nonequilibrium statistical mechanics of dividing cell populations. Phys. Rev. Lett. 99, 138102 (doi:10.1103/PhysRevLett.99.138102) 17930641 10.1103/PhysRevLett.99.138102

[26] Grima R, Schnell S 2008 Modelling reaction kinetics inside cells. Essays Biochem. 45, 41 (doi:10.1042/BSE0450041) 18793122 10.1042/BSE0450041 PMC2737326

[27] Dowman JE 1973 Implications of stochastic inheritance. J. Theor. Biol. 39, 55–72. (doi:10.1016/0022-5193(73)90205-1) 4582399 10.1016/0022-5193(73)90205-1

[28] Loeffler M, Grossmann B 1991 A stochastic branching model with formation of subunits applied to the growth of intestinal crypts. J. Theor. Biol. 150, 175–191. (doi:10.1016/S0022-5193(05)80330-3) 1890854 10.1016/s0022-5193(05)80330-3

[29] Grey D, Hutson V, Szathmary EO 1995 A re-examination of the stochastic corrector model. Proc. R. Soc. Lond. B 262, 29–35. (doi:10.1098/rspb.1995.0172)

[30] Swain PS, Elowitz MB, Siggia ED 2002 Intrinsic and extrinsic contributions to stochasticity in gene expression. Proc. Natl Acad. Sci. USA 99, 12 795–12 800. (doi:10.1073/pnas.162041399) 10.1073/pnas.162041399 PMC130539 12237400

[31] Rausenberger J, Kollmann M 2008 Quantifying origins of cell-to-cell variations in gene expression. Biophys. J. 95, 4523–4528. (doi:10.1529/biophysj.107.127035) 18689455 10.1529/biophysj.107.127035 PMC2576406

[32] Gillespie DT 1977 Exact stochastic simulation of coupled chemical reactions. J. Phys. Chem. 81, 2340–2361. (doi:10.1021/j100540a008)

[33] Cree LM, Samuels DC, de Sousa Lopes SC, Rajasimha HK, Wonnapinij P, Mann JR, Dahl HH, Chinnery PF 2008 A reduction of mitochondrial DNA molecules during embryogenesis explains the rapid segregation of genotypes. Nat. Genet. 40, 249–254. (doi:10.1038/ng.2007.63) 18223651 10.1038/ng.2007.63

[34] Cao L, Shitara H, Horii T, Nagao Y, Imai H, Abe K, Hara T, Hayashi JI, Yonekawa H 2007 The mitochondrial bottleneck occurs without reduction of mtDNA content in female mouse germ cells. Nat. Genet. 39, 386–390. (doi:10.1038/ng1970) 17293866 10.1038/ng1970

[35] Wai T, Teoli D, Shoubridge EA 2008 The mitochondrial DNA genetic bottleneck results from replication of a subpopulation of genomes. Nat. Genet. 40, 1484–1488. (doi:10.1038/ng.258) 19029901 10.1038/ng.258

[36] Wallace D, Chalkia D 2013 Mitochondrial DNA genetics and the heteroplasmy conundrum in evolution and disease. Cold Spring Harbor Perspect. Biol. 5, a021220 (doi:10.1101/cshperspect.a021220) 10.1101/cshperspect.a021220 PMC3809581 24186072

[37] Carling PJ, Cree LM, Chinnery PF 2011 The implications of mitochondrial DNA copy number regulation during embryogenesis. Mitochondrion 11, 686–692. (doi:10.1016/j.mito.2011.05.004) 21635974 10.1016/j.mito.2011.05.004

[38] Wright S 1942 Statistical genetics and evolution. Bull. Am. Math. Soc. 48, 223–247. (doi:10.1090/S0002-9904-1942-07641-5)

[39] Kimura M 1955 Solution of a process of random genetic drift with a continuous model. Proc. Natl Acad. Sci. USA 41, 144–150. (doi:10.1073/pnas.41.3.144) 16589632 10.1073/pnas.41.3.144 PMC528040

[40] Wonnapinij P, Chinnery PF, Samuels DC 2008 The distribution of mitochondrial DNA heteroplasmy due to random genetic drift. Am. J. Hum. Genet. 83, 582–593. (doi:10.1016/j.ajhg.2008.10.007) 18976726 10.1016/j.ajhg.2008.10.007 PMC2668051

[41] Iizuka M 2001 The effective size of fluctuating populations. Theor. Popul. Biol. 59, 281–286. (doi:10.1006/tpbi.2001.1521) 11560448 10.1006/tpbi.2001.1521

[42] Maruyama T, Kimura M 1980 Genetic variability and effective population size when local extinction and recolonization of subpopulations are frequent. Proc. Natl Acad. Sci. USA 77, 6710–6714. (doi:10.1073/pnas.77.11.6710) 16592920 10.1073/pnas.77.11.6710 PMC350358

[43] Johnston IG, Burgstaller J, Vitezslav H, Kolbe T, Rülicke T, Brem G, Poulton J, Jones N In press Stochastic modelling, Bayesian inference, and new in vivo measurements elucidate the debated mtDNA bottleneck mechanism. eLife. (doi:10.7554/eLife.07464) 10.7554/eLife.07464 PMC4486817 26035426

[44] Lawson K, Hage W 1994 Clonal analysis of the origin of primordial germ cells in the mouse. CIBA Foun. Symp. 165, 68–84. 10.1002/9780470514573.ch5 7835158

[45] Chinnery PF, Samuels DC 1999 Relaxed replication of mtDNA: a model with implications for the expression of disease. Am. J. Hum. Genet. 64, 1158–1165. (doi:10.1086/302311) 10090901 10.1086/302311 PMC1377840

[46] DiMauro S, Schon EA 2001 Mitochondrial DNA mutations in human disease. Am. J. Med. Genet. 106, 18–26. (doi:10.1002/ajmg.1392) 11579421 10.1002/ajmg.1392

[47] Melton T 2004 Mitochondrial DNA heteroplasmy. Forensic Sci. Rev. 16, 1–20. 26256810

[48] Capps GJ, Samuels DC, Chinnery PF 2003 A model of the nuclear control of mitochondrial DNA replication. J. Theor. Biol. 221, 565–583. (doi:10.1006/jtbi.2003.3207) 12713941 10.1006/jtbi.2003.3207

[49] Elson JL, Samuels DC, Turnbull DM, Chinnery PF 2001 Random intracellular drift explains the clonal expansion of mitochondrial DNA mutations with age. Am. J. Hum. Genet. 68, 802–806. (doi:10.1086/318801) 11179029 10.1086/318801 PMC1274494

[50] Payne BAI, Wilson IJ, Hateley CA, Horvath R, Santibanez-Koref M, Samuels DC, Price DA, Chinnery PF 2011 Mitochondrial aging is accelerated by anti-retroviral therapy through the clonal expansion of mtDNA mutations. Nat. Genet. 43, 806–810. (doi:10.1038/ng.863) 21706004 10.1038/ng.863 PMC3223397

[51] Stegun I, Abramowitz M 1972 Handbook of mathematical functions: with formulas, graphs, and mathematical tables. New York, NY: Dover.

[52] Solieri L 2010 Mitochondrial inheritance in budding yeasts: towards an integrated understanding. Trends Microbiol. 18, 521–530. (doi:10.1016/j.tim.2010.08.001) 20832322 10.1016/j.tim.2010.08.001

[53] Rafelski SM *et al* 2012 Mitochondrial network size scaling in budding yeast. Science 338, 822–824. (doi:10.1126/science.1225720) 23139336 10.1126/science.1225720 PMC3602416

[54] Nicolson GL 1987 Tumor cell instability, diversification, and progression to the metastatic phenotype: from oncogene to oncofetal expression. Cancer Res. 47, 1473–1487. 3545445

[55] Johnston IG 2014 Efficient parametric inference for stochastic biological systems with measured variability. Stat. Appl. Genet. Mol. Biol. 13, 379–390. (doi:10.1515/sagmb-2013-0061) 24821877 10.1515/sagmb-2013-0061

