# Supplementary material for: Closed-form stochastic solutions for non-equilibrium dynamics and inheritance of cellular components over many cell divisions
Source: Proc Math Phys Eng Sci. 2015 Aug 8;471(2180):20150050. doi: 10.1098/rspa.2015.0050 (PMC4550007; doi:10.1098/rspa.2015.0050)
Supplement: Appendix [file rspa20150050supp1.pdf]

Appendix

In this Appendix we present some of the lengthier mathematical results used in the main text. We include a Mathematica notebook as part of this article, containing the following derivations.

Generating function for the BID process

The derivation of the generating function for BID dynamics in the absence of cell divisions is well known: we include it here for completeness. We write the PDE describing the generating function $G(z,t)$ in Laplace form:

$$\frac{\partial G(z,t)}{dt} - (\nu(1-z) + \lambda(z^2 - z))\frac{\partial G(z,t)}{\partial z} = \alpha(z-1)G \quad (1)$$

$$G(z,0) = z^{m_0}, \quad (2)$$

$$(3)$$

We proceed by using the method of characteristics, writing down ODEs describing how the parameters of G, and G itself, changes along a characteristic curve, with progress along such a curve parameterised by s. The corresponding ODEs are

$$\frac{dt}{ds} = 1 \quad (4)$$

$$\frac{dz}{ds} = -(\nu(1-z) + \lambda(z^2 - z)) \quad (5)$$

$$\frac{dG}{ds} = \alpha(z-1)G \quad (6)$$

Eqn. 4 lets us immediately set $t = s$, omitting a constant of integration as the absolute value of progress along a characteristic curve is unimportant. Using $t = s$ throughout, Eqn. 5 is solved by

$$z = \frac{1 - \nu e^{c_1(\lambda-\nu) - t(\lambda-\nu)}}{1 - \lambda e^{c_1(\lambda-\nu) - t(\lambda-\nu)}} \quad (7)$$

where c_1 is a constant of integration, the explicit form of which will be useful later. Rearranging this into an expression for c_1 gives

$$c_1 = t + \frac{\ln\left(\frac{z-1}{\lambda z - \nu}\right)}{\lambda - \nu}. \quad (8)$$

Finally, Eqn. 6 with Eqn. 7 gives us

$$G = c_2 e^{-\alpha t}\left(e^{\lambda t + c_1 \nu} - \lambda e^{\lambda c_1 + \nu t}\right)^{\alpha/\lambda}. \quad (9)$$

c_2 is a function of c_1 because the quantity c_1, the constant of integration acquired when integrating z with respect to s, is independent of s, and hence forms an independent parameter when integrating G with respect to s. We require that $G(t=0) = z^{m_0}$, so we choose

$$c_2(c_1) = \left(e^{c_1\nu} - \lambda e^{c_1\lambda}\right)^{-\alpha/\lambda}\left(\frac{\nu e^{(\lambda-\nu)c_1} - 1}{\lambda e^{(\lambda-\nu)c_1} - 1}\right)^{m_0}, \quad (10)$$

where the first term cancels the final term in Eqn. 9 when $t = 0$, and the final term can be seen to extract a factor z^{m_0} from Eqn. 8 for c_1 when $t = 0$. We then have

$$G(z,t) = c_2(c_1(z,t))e^{-\alpha t}\left(e^{\lambda t + c_1(z,t)\nu} - \lambda e^{\lambda c_1(z,t) + \nu t}\right)^{\alpha/\lambda} \quad (11)$$

which, after inserting Eqn. 8 and some algebra, gives

$$G(z,t) = \left(\frac{\nu-\lambda}{\lambda e^{(\lambda-\nu)t}(z-1)-\lambda z+\nu}\right)^{\frac{\alpha}{\lambda}} \left(\frac{\nu e^{(\lambda-\nu)t}(z-1)-\lambda z+\nu}{\lambda e^{(\lambda-\nu)t}(z-1)-\lambda z+\nu}\right)^{m_0} \tag{12}$$

$$\equiv \xi(z,t)(g(z,t))^{m_0} \tag{13}$$

Recurrence relations arising from induction over cell divisions

This section focusses on the solution of recurrence relations of the form

$$\zeta_i = \frac{a\zeta_{i+1}+b}{c\zeta_{i+1}+d}, \text{ or equivalently, } \zeta_{i+1} = \frac{d\zeta_i - b}{-c\zeta_i + a} \tag{14}$$

In the Main Text, both h_i and z_i follow relationships of this kind; we use the symbol ζ_i here to emphasise that the same solution strategy applies in both cases, and describe specific solutions below. This system is solved, after [1], by defining $\alpha \equiv \frac{a+d}{c}$, $\beta \equiv \frac{D}{c^2}$, $D \equiv ad - bc$, $y_i = \zeta_i + \frac{d}{c}$ and implicitly defining w_i through $y_i = \frac{w_i}{w_{i+1}}$. These changes of variables allow us to find an expression for w_i, which can then be substituted back through the above chain to find ζ_i. We have

$$y_i = \alpha - \frac{\beta}{y_{i+1}} \tag{15}$$

$$\frac{w_i}{w_{i+1}} = \alpha - \frac{\beta w_{i+2}}{w_{i+1}} \tag{16}$$

$$\to \beta w_{i+2} - \alpha w_{i+1} + w_i = 0, \tag{17}$$

which is solved by considering solutions to the characteristic equation $\beta k^2 - \alpha k + 1 = 0$, which are straightforwardly $k_{1,2} = \frac{1}{2\beta}(\alpha \pm \sqrt{\alpha^2 - 4\beta})$. Then

$$w_i = C_1 k_1^i + C_2 k_2^i \tag{18}$$

$$y_i = \frac{C_0 k_1^i + k_2^i}{C_0 k_1^{i+1} + k_2^{i+1}} \tag{19}$$

$$\zeta_i = \frac{C_0 k_1^i + k_2^i}{C_0 k_1^{i+1} + k_2^{i+1}} - \frac{d}{c} \tag{20}$$

where C_i are constants to be determined from boundary conditions. If the boundary condition takes the form $\zeta_n = \frac{pz+q}{rz+s}$, as is the case throughout the situations we consider, we obtain

$$\frac{C_0 k_1^i + k_2^i}{C_0 k_1^{i+1} + k_2^{i+1}} - \frac{d}{c} = \frac{pz+q}{rz+s} \tag{21}$$

$$\Rightarrow C_0 = \frac{k_2^n k_1^{-n}(k_2 c(pz+q) + k_2 d(rz+s) - c(rz+s))}{c(rz+s) - k_1 c(pz+q) - k_1 d(rz+s)}. \tag{22}$$

Thus, given knowledge of a, b, c, d from the recurrence relation and p, q, r, s from the initial condition, we can obtain k_1, k_2 through α and β and hence use Eqns. 22 and 20 to obtain a solution to the recurrence relation. Below, we use this approach to obtain solutions for the systems of interest in the main text.

Binomial partitioning solution for h

We will use the substitutions $l = e^{(\lambda-\nu)\tau}, l' = e^{(\lambda-\nu)t}$. The original recurrence relation is

$$h_i = g\left(\frac{1}{2} + \frac{h_{i+1}}{2}, \tau\right) \tag{23}$$

$$= \frac{(\nu l - \lambda)h_{i+1} + (-\lambda - \nu(l-2))}{(\lambda(l-1))h_{i+1} + (2\nu - \lambda(l+1))} \tag{24}$$

$$h_n = g(z, t) \tag{25}$$

$$= \frac{(\nu l' - \lambda)z + (\nu - \nu l')}{(\lambda(l'-1))z + (\nu - \lambda l')}; \tag{26}$$

hence, $a = (\nu l - \lambda), b = (2\nu - \nu l - \lambda), c = (\lambda l - \lambda), d = (2\nu - \lambda - \lambda l), p = (\nu l' - \lambda), q = (\nu - \nu l'), r = (\lambda(l'-1)), s = (\nu - \lambda l')$. Using these values to determine α, k_1, k_2, and after some algebra, we obtain

$$h_i = \frac{2^i l^n l'(z-1)(\lambda + \nu(l-2)) + 2^n l^i(\lambda(l' - z(l+l'-2)) + \nu(l-2))}{2^i l^n l' \lambda(l-1)(z-1) + 2^n l^i(\lambda(l' - z(l+l'-2)) + \nu(l-2))}. \tag{27}$$

Subtractive partitioning solution for h

$$h_i = g(h_{i+1}, \tau) \tag{28}$$

$$= \frac{(\nu l - \lambda)h_{i+1} + (\nu - \nu l)}{(\lambda(l-1))h_{i+1} + (\nu - \lambda l)}; \tag{29}$$

$$h_n = g(z, t), \tag{30}$$

$$= \frac{(\nu l' - \lambda)z + (\nu - \nu l')}{(\lambda(l'-1))z + (\nu - \lambda l')}; \tag{31}$$

hence $a = (\nu l - \lambda), b = (\nu - \nu l), c = (\lambda(l-1)), d = (\nu - \lambda l), p = (\nu l' - \lambda), q = (\nu - \nu l'), r = \lambda(l'-1), s = (\nu - \lambda l')$. Then

$$h_i = \frac{l^n l' \nu(z-1) + l^i(\nu - \lambda z)}{l^n l' \lambda(z-1) + l^i(\nu - \lambda z)} \tag{32}$$

Binomial partitioning solution for z

$$z_i = \frac{1}{2} + \frac{g(z_{i-1}, \tau)}{2} \tag{33}$$

$$= \frac{(l(\lambda+\nu) - 2\lambda)z_{i-1} + (2\nu - l(\lambda+\nu))}{(2\lambda(l-1))z_{i-1} + (2\nu - 2\lambda l)} \tag{34}$$

$$\Rightarrow z_i = \frac{(2\nu - 2\lambda l)z_{i+1} + (l(\lambda+\nu) - 2\nu)}{(2\lambda(1-l))z_{i+1} + (l(\lambda+\nu) - 2\lambda)} \tag{35}$$

$$z_1 = \frac{1}{2} + \frac{g(z, t)}{2}. \tag{36}$$

$$= \frac{(l'(\lambda+\nu) - 2\lambda)z + (2\nu - l'(\lambda+\nu))}{(2\lambda(l'-1))z + (2\nu - 2\lambda l')}. \tag{37}$$

In Eqn. 35 we have used the equivalence in Eqn. 14 to rewrite the recurrence relation in the form we have previously solved. Subsequently, $a = (2\nu - 2\lambda l), b = (l(\lambda+\nu) - 2\nu), c = (2\lambda(1-l)), d = (l(\lambda+\nu) - 2\lambda), p = (l'(\lambda+\nu) - 2\lambda), q = (2\nu - l'(\lambda+\nu)), r = (2\lambda(l'-1)), s = (2\nu - 2\lambda l')$, leading to

$$z_i = \frac{l^i l'(z-1)(l(\lambda+\nu) - 2\nu) - 2^i l(\lambda l'(z-1) + (l-2)(\lambda z - \nu))}{2\lambda l^i l'(z-1)(l-1) - 2^i l(\lambda l'(z-1) + (l-2)(\lambda z - \nu))} \tag{38}$$

Subtractive partitioning solution for z

$$
\begin{aligned}
z_i &= g(z_{i-1},\tau), && (39)\\
&= \frac{(\nu l-\lambda)z_{i-1}+(\nu-\nu l)}{(\lambda(l-1))z_{i-1}+(\nu-\lambda l)}; && (40)\\
\Rightarrow z_i &= \frac{(\nu-\lambda l)z_{i+1}+(\nu l-\nu)}{(\lambda(1-l))z+(\nu l-\lambda)} && (41)\\
z_1 &= g(z,t), && (42)\\
&= \frac{(\nu l'-\lambda)z+(\nu-\nu l')}{(\lambda(l'-1))z+(\nu-\lambda l')}; && (43)
\end{aligned}
$$

Hence $a=(\nu-\lambda l), b=(\nu l-\nu), c=(\lambda(1-l)), d=(\nu l-\lambda), p=(\nu l'-\lambda), q=(\nu-\nu l'), r=\lambda(l'-1), s=(\nu-\lambda l')$, leading to

$$z_i = \frac{l^i l' \nu(z-1)+l(\nu-\lambda z)}{l^i l' \lambda(z-1)+l(\nu-\lambda z)} \tag{44}$$

Products of prefactors over the inductive process

We are concerned with an expression for the product $\prod_{i=1}^{n}\phi(z_i,\tau)$ from the Main Text. We first consider $\prod_{i=1}^{n}\xi(z_i,\tau)$ which occurs as a factor in this expression in every partitioning regime. We recall that $\xi(z,t)$ has the form

$$\xi(z,t) = \left(\frac{\nu-\lambda}{\lambda e^{(\lambda-\nu)t}(z-1)-\lambda z+\nu}\right)^{\alpha/\lambda} \tag{45}$$

If we write z_i in the form

$$z_i = \frac{\tilde{A}_1\rho_A^i+\tilde{B}_1\rho_B^i}{\tilde{A}_2\rho_A^i+\tilde{B}_2\rho_B^i}, \tag{46}$$

a general form separating variables raised to the power i in z_i (here represented by ρ), from their coefficients $\tilde{A}_j,\tilde{B}_j$, the form of Eqn. 45 gives us:

$$\xi(z_i,\tau) = \left(\frac{A_1\rho_A^i+B_1\rho_B^i}{A_2\rho_A^i+B_2\rho_B^i}\right)^{\gamma}, \tag{47}$$

where $A_1=(\tilde{A}_2(\nu-\lambda)), B_1=(\tilde{B}_2(\nu-\lambda)), A_2=(\lambda l(\tilde{A}_1-\tilde{A}_2)+\nu\tilde{A}_2-\lambda\tilde{A}_1), B_2=(\lambda l(\tilde{B}_1-\tilde{B}_2)+\nu\tilde{B}_2-\lambda\tilde{B}_1)$, $\gamma=\alpha/\lambda$, following from the form of $\xi(z,t)$.

The product of an expression of this form can be written

$$\prod_{i=1}^{n}\xi(z_i,t_i) \equiv \prod_{i=1}^{n}\left(\frac{A_1\rho_A^i+B_1\rho_B^i}{A_2\rho_A^i+B_2\rho_B^i}\right)^{\gamma} = \left(B_1^n\rho_B^{n(n+1)/2}\prod_{i=1}^{n}\left(1+\frac{A_1}{B_1}\frac{\rho_A^i}{\rho_B^i}\right)\right)^{\gamma}\left(B_2^n\rho_B^{n(n+1)/2}\prod_{i=1}^{n}\left(1+\frac{A_2}{B_2}\frac{\rho_A^i}{\rho_B^i}\right)\right)^{-\gamma} \tag{48}$$

Here we make use of the q-Pochhammer symbol $(a;q)_n$, defined by

$$(a;q)_n \equiv \prod_{k=0}^{n-1}(1-aq^k). \tag{49}$$

The terms within the brackets on the RHS of Eqn. 48 then become, by setting $a = -A_j/B_j$ and $q = \rho_A/\rho_B$,

$$B_j^n \rho_B^{n(n+1)/2} \prod_{i=1}^{n} \left(1 + \frac{A_j}{B_j}\frac{\rho_A^i}{\rho_B^i}\right) \equiv B_j^n \rho_B^{n(n+1)/2} \frac{1}{1 + A_j/B_j}(-A_j/B_j; \rho_A/\rho_B)_{n+1}, \quad (50)$$

$$= B_j^{n+1} \rho_B^{n(n+1)/2} \frac{(-A_j/B_j; \rho_A/\rho_B)_{n+1}}{A_j + B_j} \quad (51)$$

and so

$$\left(B_1^n \rho_B^{n(n+1)/2} \prod_{i=1}^{n} \left(1 + \frac{A_1}{B_1}\frac{\rho_A}{\rho_B}\right)\right)^{\gamma} \left(B_2^n \rho_B^{n(n+1)/2} \prod_{i=1}^{n} \left(1 + \frac{A_2}{B_2}\frac{\rho_A}{\rho_B}\right)\right)^{-\gamma} \equiv \left(\frac{B_1^{n+1} B_2^{-n-1}(A_2 + B_2)(-A_1/B_1; \rho_A/\rho_B)_{n+1}}{(A_1 + B_1)(-A_2/B_2; \rho_A/\rho_B)_{n+1}}\right)^{\gamma}, \quad (52)$$

yielding a simple form for the product of interest. For the binomial case, we have from Eqn. 38:

$$z_i = \frac{l^i l'(z-1)(l(\lambda+\nu) - 2\nu) - 2^i l(\lambda l'(z-1) + (l-2)(\lambda z - \nu))}{2\lambda l^i l'(z-1)(l-1) - 2^i l(\lambda l'(z-1) + (l-2)(\lambda z - \nu))}. \quad (53)$$

Here, the two quantities raised to the power of i in z_i are l and 2, so we set $\rho_A \equiv l$, $\rho_b \equiv 2$. Then by comparing coefficients we identify $\tilde{A}_1 = (l'(z-1)(l(\lambda+\nu)-2\nu))$, $\tilde{B}_1 = (-l(\lambda l'(z-1)+(l-2)(\lambda z-\nu)))$, $\tilde{A}_2 = (2\lambda l'(z-1)(l-1))$, $\tilde{B}_2 = (-l(\lambda l'(z-1) + (l-2)(\lambda z - \nu)))$, hence $A_1 = 2\lambda l'(l-1)(z-1)(\nu - \lambda)$, $A_2 = \lambda l l'(l-1)(z-1)(\nu - \lambda)$, $B_1 = B_2 = -l(\lambda l'(z-1) + (l-2)(\lambda z - \nu))(\nu - \lambda)$, and finally $\gamma = \alpha/\lambda$.

For the subtractive case, we have from Eqn. 44:

$$z_i = \frac{l^i l' \nu(z-1) + l(\nu - \lambda z)}{l^i l' \lambda(z-1) + l(\nu - \lambda z)}. \quad (54)$$

Now the quantities raised to the power of i are l and 1, so we set $\rho_A = l, \rho_B = 1$, and find $\tilde{A}_1 = (l'\nu(z-1))$, $\tilde{B}_1 = (l(\nu - \lambda z))$, $\tilde{A}_2 = (l'\lambda(z-1))$, $\tilde{B}_2 = (l(\nu - \lambda z))$, hence $A_1 = \lambda l'(z-1)(\nu - \lambda)$, $A_2 = \lambda l l'(z-1)(\nu - \lambda)$, $B_1 = B_2 = l(\nu - \lambda)(\nu - \lambda z)$, and $\gamma = \alpha/\lambda$.

Other products

We can also use this result to compute the product of exponentiated prefactors involved in the subtractive inheritance regimes. We will first consider the product $\prod_{i=1}^{n} g(z_i, \tau)^{-\eta}$, which plays a role in the deterministic subtractive inheritance we consider later. We recall the definitions of the recurrence relations in the Main Text

$$h_i(z,t) = g(\theta(h_{i+1}), \tau) \; ; \; h_n(z,t) = g(z,t) \quad (55)$$

$$z_{i+1} = \theta(g(z_i, \tau)) \; ; \; z_1 = \theta(g(z,t)). \quad (56)$$

For both subtractive inheritance cases, $\theta(g(z,t)) = g(z,t)$, so it can straightforwardly be seen that

$$z_{i+1} = g(z_i, \tau) \; ; \; z_1 = g(z,t) \quad (57)$$

$$h_i(z,t) = g(h_{i+1}, \tau) \; ; \; h_n(z,t) = g(z,t) \quad (58)$$

$$\text{and so } h_n(z,t) = z_1 \; ; \; h_{n-i+1} = z_i. \quad (59)$$

Thus, the product $\prod_{i=1}^{n} g(z_i, \tau)^{-\eta}$ is equivalent to the product $\prod_{j=1}^{n} h_j^{-\eta}$, where $j = n - i + 1$. As i and j are dummy variables, we can then identify the required solution as $\prod_{i=1}^{n} h_i^{-\eta}$. We have from Eqn. 32 that

$$h_i = \frac{l^n l' \nu(z-1) + l^i(\nu - \lambda z)}{l^n l' \lambda(z-1) + l^i(\nu - \lambda z)} \quad (60)$$

$$= \frac{\nu l' x_1^i (-x_2)^n (z-1) + x_1^n (-x_2)^i (\nu - \lambda z)}{\lambda l' x_1^i (-x_2)^n (z-1) + x_1^n (-x_2)^i (\nu - \lambda z)} \quad (61)$$

where we have rewritten the final line to avoid a diverging factor of $(z-1)^{-1}$ appearing in the Pochhammer symbol, using

$$\begin{aligned} x_1 &= \frac{\lambda}{\lambda-\nu}(e^{(\nu-\lambda)\tau}-1) && (62)\\ x_2 &= \frac{\lambda}{\lambda-\nu}(e^{(\lambda-\nu)\tau}-1) && (63)\end{aligned}$$

We then see that $h_i^{-\eta}$ is of the form Eqn. 47, so we can use the result therein, with $A_1 = \nu l'(-x_2)^n(z-1), B_1 = B_2 = x_1^n(\nu-\lambda z), A_2 = \lambda l'(-x_2)^n(z-1), \rho_A = x_1, \rho_B = (-x_2), \gamma = -\eta$.

Next, we wish to compute an expression for $\prod_{i=1}^n \left(\frac{1}{2} + \frac{1}{2g(z_i,t)}\right)^{2\eta}$, of use in the random subtractive regime. We again exploit the relation between $g(z_i,\tau)$ and h_j in Eqn. 59 to show that the kernel of the desired product is equivalent to $\left(\frac{1}{2}+\frac{1}{2h_i}\right)$. Again, we use h_i from Eqn. 32; after some algebra this expression reduces to

$$\frac{l'x_1^i(-x_2)^n(z-1)(\lambda+\nu)+2x_1^n(-x_2)^i(\nu-\lambda z)}{2l'x_1^i(-x_2)^n\nu(z-1)+2x_1^n(-x_2)^i(\nu-\lambda z)}, \tag{64}$$

whereupon we can use Eqn. 52 with $A_1 = l'(-x_2)^n(z-1)(\lambda+\nu), B_1 = B_2 = 2x_1^n(\nu-\lambda z), A_2 = 2l'(-x_2)^n\nu(z-1), \rho_A = x_1, \rho_B = (-x_2), \gamma = 2\eta$.

Full forms of generating functions

To recap, we use α, λ, ν to respectively represent the rates of immigration, birth, and death in our model; m_0 for initial copy number; τ for cell cycle length, n for the number of divisions that have occurred, and t for the elapsed time since the most recent cell division. We employ simplifying symbols $l \equiv e^{(\lambda-\nu)\tau}; l' \equiv e^{(\lambda-\nu)t}$ and $x_1 \equiv \lambda(l^{-1}-1)/(\lambda-\nu); x_2 \equiv \lambda(l-1)/(\lambda-\nu)$.

The general form of the generating functions was shown in the Main Text to be

$$G_n(z,t) = \underbrace{\left(\prod_{i=1}^n \xi(z_i,\tau)\right)}_{\text{(i)}} \underbrace{\left(\prod_{i=1}^n \phi(z_i,\tau)\right)}_{\text{(ii)}} \underbrace{\xi(z,t)}_{\text{(iii)}} \underbrace{h_0(z,t)^{m_0}}_{\text{(iv)}}, \tag{65}$$

where z_i and h_i are the solutions to recursion relations defined in the Main Text. The term (iii) is the same in all calculations and is, from Eqn. 12,

$$\xi(z,t) = \left(\frac{\nu-\lambda}{\lambda l(z-1)-\lambda z+\nu}\right)^{\alpha/\lambda}. \tag{66}$$

The generating function in the case of binomial partitioning at cell divisions involves (i) Eqn. 52 applied to the appropriate terms described in Eqn. 53; (ii) unity; (iii) Eqn. 66; and (iv) h_0 from Eqn. 27, giving overall

$$\begin{aligned} G_n(z,t) &= \left(\frac{(\lambda l l'(l-1)(z-1)-l(\lambda l'(z-1)+(l-2)(\lambda z-\nu)))\left(\frac{-2\lambda l'(l-1)(z-1)}{-l(\lambda l'(z-1)+(l-2)(\lambda z-\nu))};\frac{l}{2}\right)_{n+1}}{(2\lambda l'(l-1)(z-1)-l(\lambda l'(z-1)+(l-2)(\lambda z-\nu)))\left(\frac{-\lambda l l'(l-1)(z-1)}{-l(\lambda l'(z-1)+(l-2)(\lambda z-\nu))};\frac{l}{2}\right)_{n+1}}\right)^{\alpha/\lambda} \\ &\times \left(\frac{\nu-\lambda}{\lambda l(z-1)-\lambda z+\nu}\right)^{\alpha/\lambda} \\ &\times \left(\frac{l^n l'(z-1)(\lambda+\nu(l-2))+2^n(\lambda(l'-z(l+l'-2))+\nu(l-2))}{l^n l'(z-1)\lambda(l-1)+2^n(\lambda(l'-z(l+l'-2))+\nu(l-2))}\right)^{m_0} \end{aligned} \tag{67}$$

The generating function in the case of random subtractive inheritance involves (i) Eqn. 52 applied to the appropriate terms described in Eqn. 54; (ii) Eqn. 52 applied to Eqn. 64 as described; (iii) Eqn. 66; and (iv) h_0 from Eqn. 32, and is

$$
\begin{aligned}
G_n(z,t) \;=\; & \left(\frac{(2l'\nu(-x_2)^n(z-1) - 2x_1^n(\lambda z-\nu)) \left(\frac{-l'(\lambda+\nu)(-x_2)^n(z-1)}{-2x_1^n(\lambda z-\nu)}; \frac{x_1}{-x_2}\right)_{n+1}}{(l'(\lambda+\nu)(-x_2)^n(z-1) - 2x_1^n(\lambda z-\nu)) \left(\frac{2l'\nu(-x_2)^n(z-1)}{-2x_1^n(\lambda z-\nu)}; \frac{x_1}{-x_2}\right)_{n+1}} \right)^{2\eta} \\
& \times \left(\frac{(\lambda l' l(z-1) + l(\nu-\lambda z)) \left(\frac{-\lambda l'(z-1)}{l(\nu-\lambda z)}; l\right)_{n+1}}{(\lambda l'(z-1) + l(\nu-\lambda z)) \left(\frac{-\lambda l' l(z-1)}{l(\nu-\lambda z)}; l\right)_{n+1}} \right)^{\alpha/\lambda} \\
& \times \left(\frac{\nu-\lambda}{\lambda l(z-1) - \lambda z + \nu} \right)^{\alpha/\lambda} \left(\frac{l^n l' \nu(z-1) + \nu - \lambda z}{l^n l' \lambda(z-1) + \nu - \lambda z} \right)^{m_0} \qquad (68)
\end{aligned}
$$

Birth-death dynamics for the mtDNA bottleneck

For birth-death dynamics, $\alpha = 0$, so for binomial partitioning the generating function takes the form of the final term in Eqn. 67.

In the case of balanced copy number, with $\lambda = \ln 2/\tau + \nu$,

$$
g(z,t) = \frac{(2^{t/\tau}-1)(z-1)\nu\tau - z\ln 2}{2^{t/\tau}(z-1)(\nu\tau + \ln 2) - z\ln 2 - \nu\tau(z-1)} \qquad (69)
$$

The corresponding solutions for z_i, h_i are

$$
z_i \;=\; \frac{2^{t/\tau}(z-1)((i+1)\nu\tau + i\ln 2) - 2(z-1)\nu\tau - z\ln 4}{2^{t/\tau}(z-1)(i+1)(\nu\tau + \ln 2) - 2(z-1)\nu\tau - z\ln 4} \qquad (70)
$$

$$
h_i \;=\; \frac{2^{t/\tau}(z-1)((i-n-2)\nu\tau + (i-n)\ln 2) + 2\nu\tau(z-1) + z\ln 4}{2^{t/\tau}(z-1)(i-n-2)(\nu\tau + \ln 2) + 2\nu\tau(z-1) + z\ln 4} \qquad (71)
$$

so

$$
G_n(z,t) = \underbrace{1}_{(i)} \times \underbrace{1}_{(ii)} \times \underbrace{1}_{(iii)} \times \underbrace{\left(\frac{2\nu\tau(z-1) - 2^{t/\tau}(z-1)((n+2)\nu\tau + n\ln 2) + z\ln 4}{2\nu\tau(z-1) - 2^{t/\tau}(z-1)(n+2)(\nu\tau + \ln 2) + z\ln 4} \right)^{m_0}}_{(iv)}. \qquad (72)
$$

Relaxed replication of mtDNA dynamics

The generating function for a single cell cycle (involving immigration and death dynamics) can straightforwardly be found

$$
G(z,t) = \exp\left((1-e^{-\beta t})m_{opt}(z-1)\right)\left(1 + e^{-\beta t}(z-1)\right)^{m_0}, \qquad (73)
$$

and so

$$
\xi(z,t) \;\equiv\; \exp\left((1-e^{-\beta t})m_{opt}(z-1)\right) \qquad (74)
$$

$$
g(z,t) \;\equiv\; 1 + e^{-\beta t}(z-1). \qquad (75)
$$

For binomial partitioning, we have $\phi(z,t) = 1$ and $\theta(g(z,t)) = (1/2 + g(z,t)/2)$. The solutions of the appropriate recurrence relations are then:

$$z_i = 1 + 2^{-i} e^{-\beta(t+(i-1)\tau)}(z-1) \tag{76}$$
$$h_i = 1 + 2^{i-n} e^{-\beta(t+(n-i)\tau)}(z-1) \tag{77}$$

So the overall generating function is

$$G_n(z,t) = \underbrace{\exp\left(m_{opt}(z-1)\left(\frac{2^{-n}e^{-\beta t}(e^{\beta\tau}-1)(2^n - e^{-\beta n\tau})}{2e^{\beta\tau}-1}\right)\right)}_{\text{(i)}} \underbrace{\times 1 \times}_{\text{(ii)}} \underbrace{\exp\left((1-e^{-\beta t})m_{opt}(z-1)\right)}_{(iii)} \times \underbrace{\left(1 + 2^{-n}e^{-\beta(t+n\tau)}(z-1)\right)^{m_0}}_{(iv)}, \tag{78}$$

which after some simplification can be written as

$$G(z,t) = e^{az+b}(cz+d)^{m_0}, \tag{79}$$

with

$$a = m_{opt}\left(1 - e^{-\beta t} + \frac{2^{-n}e^{-\beta t}(e^{\beta\tau}-1)(2^n - e^{-\beta n\tau})}{2e^{\beta\tau}-1}\right); \tag{80}$$
$$b = -a; \tag{81}$$
$$c = 2^{-n}e^{-\beta(t+n\tau)}; \tag{82}$$
$$d = 1 - c. \tag{83}$$

In particular, the mean copy number is $\left(ae^{az+b}(cz+d)^{m_0} + e^{az+b}m_0 c(cz+d)^{m_0-1}\right)_{z=1}$, giving

$$\mathbb{E}(m,t) = 2^{-n}e^{-\beta(t+n\tau)}m_0 + m_{opt}\left(1 - e^{-\beta t} + \frac{2^{-n}e^{-\beta t}(e^{\beta\tau}-1)(2^n - e^{-\beta n\tau})}{2e^{\beta\tau}-1}\right) \tag{84}$$

and setting $t = \tau$ (at the end of a cell cycle), we obtain

$$\mathbb{E}(m,\tau) - m_{opt} = \frac{1 + 2^{-n}(e^{-\beta n\tau} - e^{-\beta(n+1)\tau})}{2e^{\beta\tau}-1} m_{opt} - 2^{-n}e^{-\beta\tau(n+1)}m_0. \tag{85}$$

The $n \to \infty$ limit of this expression is

$$\mathbb{E}(m,\tau) - m_{opt} \xrightarrow{n\to\infty} \frac{1}{2e^{\beta\tau}-1} m_{opt}. \tag{86}$$

In this $n \to \infty$ limit the generating function reduces to

$$G(z,t) \xrightarrow{n\to\infty} \exp\left(m_{opt}(z-1)\left(1 - e^{-\beta t} + \frac{e^{-\beta t}(e^{-\beta t}-1)}{2e^{\beta\tau}-1}\right)\right), \tag{87}$$

so that

$$P(m,t) \xrightarrow{n\to\infty} \frac{1}{m!}\left(\frac{m_{opt}(1 - 2e^{\beta\tau} + e^{-\beta(t-\tau)})}{1 - 2e^{\beta\tau}}\right)^m \exp\left(\frac{-m_{opt}(1 - 2e^{\beta\tau} + e^{-\beta(t-\tau)})}{1 - 2e^{\beta\tau}}\right). \tag{88}$$

Using Eqn. 79 and Leibniz's rule, the general probability distribution function is given by

$$
\begin{aligned}
P(m,t) &= \frac{1}{m!}\frac{\partial^m G}{\partial z^m}\bigg|_{z=0} && (89)\\
&= \frac{1}{m!}\sum_{k=0}^{m}\binom{m}{k}a^{m-k}e^{az+b}\frac{m_0!}{(m_0-k)!}(cz+d)^{m_0-k}\bigg|_{z=0} && (90)\\
&= \frac{e^{az+b}(-c)^m(cz+d)^{m_0-m}}{m!}U\left(-m,1-m+m_0,\frac{-a(cz+d)}{c}\right)\bigg|_{z=0}, && (91)\\
&= (1/m!)(-c)^m d^{m_0-m}e^b U\left(-m,1-m+m_0,-ad/c\right) && (92)
\end{aligned}
$$

where $U(a,b,z)$ is the confluent hypergeometric function.

For subtractive partitioning, we have $\phi(z,t)=(1/2+1/(2g(z,t)))^{2\eta}$ and $\theta(g(z,t))=g(z,t)$. The solutions of the appropriate recurrence relations are then:

$$
\begin{aligned}
z_i &= 1+e^{-\beta(t+(i-1)\tau)}(z-1) && (93)\\
h_i &= 1+e^{-\beta(t+(n-i)\tau)}(z-1) && (94)
\end{aligned}
$$

So the overall generating function is

$$
\begin{aligned}
G_n(z,t) &= \underbrace{\exp\left(m_{opt}(z-1)\left(e^{-\beta t}-e^{-\beta(t+n\tau)}\right)\right)}_{(i)}\underbrace{4^{\eta}\left(\frac{\left(z-1+e^{\beta(t+n\tau)}\right)\left(\frac{-1}{2}e^{-\beta(t+n\tau)}(z-1);e^{\beta\tau}\right)_{n+1}}{\left(z-1+2e^{\beta(t+n\tau)}\right)\left(-e^{-\beta(t+n\tau)}(z-1);e^{\beta\tau}\right)_{n+1}}\right)^{2\eta}}_{(ii)}\\
&\times\underbrace{\exp\left(m_{opt}(z-1)(1-e^{-\beta t})\right)}_{(iii)}\underbrace{\left(1+e^{-\beta(t+n\tau)}(z-1)\right)^{m_0}}_{(iv)}. && (95)
\end{aligned}
$$

In particular,

$$
\mathbb{E}(m,t)=m_{opt}+e^{-\beta(t+n\tau)}(m_0-m_{opt}+\eta(1+(0;e^{\beta\tau})'_{n+1})). \quad (96)
$$

It follows straightforwardly from the definition of the q-Pochhammer symbol that

$$
\begin{aligned}
(0;q)'_{n+1} &\equiv \frac{q^{n+1}-1}{q-1} && (97)\\
(0;q)''_{n+1} &\equiv q\frac{(q^n-1)(q^{n+1}-1)}{(q+1)(q-1)^2} && (98)
\end{aligned}
$$

Using these results and setting $t=\tau$ (at the end of a cell cycle), we obtain after some manipulation

$$
\mathbb{E}(m,\tau)-m_{opt}=m_0e^{-\beta\tau(n+1)}+\frac{\eta(1-e^{-\beta n\tau})+m_{opt}(e^{-\beta(n+1)\tau}+e^{-\beta n\tau})}{1-e^{\beta\tau}} \quad (99)
$$

and the only term retained in the $n\to\infty$ limit is

$$
\mathbb{E}(m,\tau)-m_{opt}\xrightarrow{n\to\infty}\frac{\eta}{1-e^{\beta\tau}}. \quad (100)
$$

Different dynamic phases

We are concerned with the extension of the generating function for the birth-death process over n cell divisions with the same rate parameters λ, ν to the case where we have different dynamic phases described by parameters $\{\lambda_1, \nu_1\}, \{\lambda_2, \nu_2\}, ...$ The generating function for the birth-death process, without immigration, is the final term in Eqn. 67. We will write this expression in the form

$$G(z,t|m_0) = \left(\frac{Pz+Q}{Rz+S}\right)^{m_0}, \tag{101}$$

with coefficients

$$\begin{aligned} P &= 2^n\lambda(l+l'-2) - l^n l'(\lambda+\nu(l-2)) & (102)\\ Q &= l^n l'(\lambda+\nu(l-2)) - 2^n(\lambda l'+\nu(l-2)) & (103)\\ R &= -\lambda l^n l'(l-1) + 2^n\lambda(l+l'-2) & (104)\\ S &= 2\lambda l^n l'(l-1) - 2^n l(\lambda l'+\nu(l-2)). & (105) \end{aligned}$$

If we now label these coefficients with an index r denoting the appropriate dynamic phase, so that, for example, P_r is Eqn. 102 with λ_r, ν_r, n_r replacing λ, ν, n, we can write:

$$\begin{aligned} h_{r_{max}} &= \frac{P_{r_{max}}z + Q_{r_{max}}}{R_{r_{max}}z + S_{r_{max}}} & (106)\\ h_r &= \frac{P_r h_{r+1} + Q_r}{R_r h_{r+1} + S_r} & (107)\\ g_{overall} = h_0 &\equiv \frac{\tilde{P}_0 z + \tilde{Q}_0}{\tilde{R}_0 z + \tilde{S}_0}, & (108) \end{aligned}$$

where $\tilde{P}_r, ...\tilde{S}_r$ are given by the recurrence equations

$$\begin{aligned} \tilde{P}_r &= P_r\tilde{P}_{r+1} + Q_r\tilde{R}_{r+1} & (109)\\ \tilde{Q}_r &= Q_r\tilde{Q}_{r+1} + Q_r\tilde{S}_{r+1} & (110)\\ \tilde{R}_r &= R_r\tilde{P}_{r+1} + S_r\tilde{R}_{r+1} & (111)\\ \tilde{S}_r &= S_r\tilde{Q}_{r+1} + S_r\tilde{S}_{r+1}, & (112) \end{aligned}$$

with $\tilde{P}_n = P_n, ..., \tilde{S}_n = S_n$. If we write the matrix

$$\mathbf{M}_r = \begin{pmatrix} P_r & Q_r \\ R_r & S_r \end{pmatrix} \tag{113}$$

the general solutions to these equations can compactly be given by

$$\begin{pmatrix} \tilde{P}_0 & \tilde{Q}_0 \\ \tilde{R}_0 & \tilde{S}_0 \end{pmatrix} = \prod_{j=0}^{r_{max}} \mathbf{M}_j \tag{114}$$

Birth-death-binomial extinction probabilities without balance and/or cell divisions

The birth-death-binomial generating function is given by setting $\alpha = 0$ in Eqn. 67. We set $\lambda = \kappa + \nu + \ln 2/\tau$ and only consider times immediately after cell divisions, hence setting $t = 0$ and $l' = 1$, giving

$$G_{BD}(z,0) = \left(\frac{2\nu(l_k-1) + \tau^{-1} l_k^n (z-1)(\kappa\tau + \nu\tau(2l_k-1) + \ln 2) + (1+z-2l_k z)(\kappa+\nu+\ln 2/\tau)}{2\nu(l_k-1) + \tau^{-1} l_k^n (z-1)(2l_k-1)(\kappa\tau + \nu\tau + \ln 2) + (1+z-2l_k z)(\kappa+\nu+\ln 2/\tau)} \right)^{m_0}, \tag{115}$$

where $l_k \equiv e^{\kappa\tau}$. Extinction probability is thus

$$P_{BD}(m=0) = \left(\frac{(l_k^n-1)(\kappa\tau + \nu\tau(2l_k-1) + \ln 2)}{\kappa\tau(2l_k^{n+1} - l_k^n - 1) + \nu\tau(2l_k-1)(l_k^n-1) - \ln 2 - l_k^n \ln 2 + l_k^{n+1}\ln 4} \right)^{m_0} \tag{116}$$

which reduces to

$$P_{BD}(m=0) = \left(\frac{1-l_k^n}{1+l_k^n - 2l_k^{n+1}} \right)^{m_0} \tag{117}$$

for $\nu = 0$, as given in the Main Text.

In the absence of cell divisions, the birth-death generating function is simply Eqn. 12 with $\alpha = 0$. Setting $z = 0$ gives the general extinction probability

$$P_{BD'}(m=0) = \left(\frac{\nu e^{(\lambda-\nu)t} - \nu}{\lambda e^{(\lambda-\nu)t} - \nu} \right)^{m_0}. \tag{118}$$

Copy number balance can be enforced in the absence of cell divisions by taking the $\lambda = \nu$ limit, from which follows the generating function

$$G_{BD',H}(z,t) = \left(\frac{z + \nu t - \nu z t}{1 + \nu t - \nu z t} \right)^{m_0} \tag{119}$$

from which straightforwardly follows the extinction probability

$$P_{BD',H}(m=0) = \left(\frac{\nu t}{1+\nu t} \right)^{m_0} \tag{120}$$

Other partitioning regimes

We have derived results for the case where a binomially-distributed random number of agents is lost at each cell division. We now consider the case where this number is a fixed constant. We will denote this constant loss number by η. In this case,

$$P_\delta(m_{i,a}|m_{i,b}) = \delta_{m_{i,a}, m_{i,b}-\eta}; \tag{121}$$

$$\sum_{m_{i,a}=0}^{m_{i,b}} \xi(z,t) \; [g(z,t)]^{m_{i,a}} \, P_\delta(m_{i,a}|m_{i,b})$$

$$= \xi(z,t) g(z,t)^{-\eta} g(z,t)^{m_{i,b}}; \tag{122}$$

$$\text{and so } \phi(z,t) = g(z,t)^{-\eta}; \tag{123}$$

$$\theta(g(z,t)) = g(z,t). \tag{124}$$

As θ, ξ and g take the same form as for the random loss case, the solutions for z_i and h_i are the same as before. The difference (due to the different form of ϕ) is in the second product in the general generating function form, which is now $\prod_{i=1}^n g(z_i,\tau)^{-\eta}$. By Eqn. 61 we have that this factor takes the form of Eqn. 52, with $A_1 = \nu l(z-1)(-x_2)^n, B_1 = B_2 = x_1^n(\nu - \lambda z), A_2 = \lambda l(z-1)(-x_2)^n, \rho_A = x_1, \rho_B = (-x_2), \gamma = -\eta$.

The generating function in the case of deterministic subtractive inheritance involves (i) Eqn. 52 applied to the appropriate terms described in Eqn. 54; (ii) Eqn. 52 applied to Eqn. 61 as described; (iii) Eqn. 66; and (iv) h_0 from Eqn. 32, and is

$$G_n(z,t) = \left(\frac{(\lambda l'(z-1)(-x_2)^n + x_1^n(\nu - \lambda z)) \left(\frac{-\nu l'(z-1)(-x_2)^n}{x_1^n(\nu-\lambda z)}; \frac{x_1}{-x_2} \right)_{n+1}}{(\nu l'(z-1)(-x_2)^n + x_1^n(\nu - \lambda z)) \left(\frac{-\lambda l'(z-1)(-x_2)^n}{x_1^n(\nu-\lambda z)}; \frac{x_1}{-x_2} \right)_{n+1}} \right)^{-\eta}$$
$$\times \left(\frac{(\lambda l' l(z-1) + l(\nu - \lambda z)) \left(\frac{-\lambda l'(z-1)}{l(\nu-\lambda z)}; l \right)_{n+1}}{(\lambda l'(z-1) + l(\nu - \lambda z)) \left(\frac{-\lambda l' l(z-1)}{l(\nu-\lambda z)}; l \right)_{n+1}} \right)^{\alpha/\lambda}$$
$$\times \left(\frac{\nu - \lambda}{\lambda l(z-1) - \lambda z + \nu} \right)^{\alpha/\lambda} \left(\frac{l^n l' \nu(z-1) + \nu - \lambda z}{l^n l' \lambda(z-1) + \nu - \lambda z} \right)^{m_0} \tag{125}$$

Now we briefly explore two other inheritance regimes of potential biological applicability. In these cases we have not been able to obtain closed-form solutions for an arbitrarily large number of cell divisions: however, the appropriate recursion relations may be followed for as many divisions as required in order to obtain a closed-form solution for the generating function.

First we consider deterministic partitioning of agents, where each daughter inherits exactly half of a parent's population. In this case:

$$P_\delta(m_{i,a}|m_{i,b}) = \delta_{m_{i,a}, m_{i,b}/2}; \tag{126}$$
$$\sum_{m_{i,a}=0}^{m_{i,b}} \xi(z,t) \; [g(z,t)]^{m_{i,a}} P_\delta(m_{i,a}|m_{i,b})$$
$$= \xi(z,t) g(z,t)^{m_{i,b}/2}; \tag{127}$$
$$\text{and so } \phi(z,t) = 1; \tag{128}$$
$$\theta(g(z,t)) = \sqrt{g(z,t)}, \tag{129}$$

leading to the recurrence relations

$$z_i = \sqrt{g(z_{i-1}, \tau)}; \; z_1 = \sqrt{g(z,t)}. \tag{130}$$
$$h_i = g\left(\sqrt{h_{i+1}}, \tau\right); \; h_n = g(z,t). \tag{131}$$

Next we consider the binomial inheritance of clusters of agents. We will assume that these clusters are of fixed size n_c. In this case, we consider the new variables $C_b = m_{i,b}/n_c$, $C_a = m_{i,a}/n_c$ (denoting the number of clusters before and after a cell division), and write

$$\sum_{m_{i,b}=0}^{\infty} \sum_{m_{i,a}=0}^{m_{i,b}} g^{m_{i,a}} P_\delta(m_{i,a}|m_{i,b}) P_{i-1}(m_{i,b}, \tau|m_0)$$
$$= \sum_{C_b=0}^{\infty} \sum_{C_a=0}^{C_b} g^{n_c C_a} \binom{C_b}{C_a} 2^{-C_b} P_{i-1}(n_c C_b, \tau|m_0) \tag{132}$$
$$= \sum_{C_b=0}^{\infty} \left(\frac{1}{2} + \frac{g^{n_c}}{2} \right)^{C_b} P_{i-1}(n_c C_b, \tau|m_0) \tag{133}$$
$$= \sum_{m_{i,b}=0}^{\infty} \left(\frac{1}{2} + \frac{g^{n_c}}{2} \right)^{\frac{m_{i,b}}{n_c}} P_{i-1}(m_{i,b}, \tau|m_0) \tag{134}$$

The resultant generating function analysis yields a very similar outcome to those previously considered, with the altered recurrence relation

$$z_i = \left(\frac{1}{2} + \frac{g(z_{i-1},\tau)^{n_c}}{2}\right)^{\frac{1}{n_c}} ; z_1 = \left(\frac{1}{2} + \frac{g(z,t)^{n_c}}{2}\right)^{\frac{1}{n_c}} \tag{135}$$

$$h_i = g_0\left(\left(\frac{1}{2} + \frac{h_{i+1}^{n_c}}{2}\right)^{\frac{1}{n_c}}, \tau\right) ; h_n = g_0(z,t). \tag{136}$$

We have been unable to reduce the recurrence relations Eqns. 130-131 or Eqns. 135-136 to a closed-form solution for birth-death dynamics, but the corresponding problems may be solved for an arbitrary number of cell divisions by writing out the recurrence explicitly, thereby obtaining the generating function for a given number of cell divisions. The figure in the main text uses this approach.

[1] L. Brand. A sequence defined by a difference equation. *Am. Math. Mon.*, 62(7):489, 1955.
